# Gradient Rotating Magnetic Fields Impairing F-Actin-Related Gene CCDC150 to Inhibit Triple-Negative Breast Cancer Metastasis by Inactivating TGF-β1/SMAD3 Signaling Pathway

**DOI:** 10.34133/research.0320

**Published:** 2024-02-28

**Authors:** Ge Zhang, Tongyao Yu, Xiaoxia Chai, Shilong Zhang, Jie Liu, Yan Zhou, Dachuan Yin, Chenyan Zhang

**Affiliations:** Institute for Special Environmental Biophysics, Key Laboratory for Space Bioscience and Biotechnology, School of Life Sciences, Northwestern Polytechnical University, 710072 Xi’an, China.

## Abstract

Triple-negative breast cancer (TNBC) is the most aggressive and lethal malignancy in women, with a lack of effective targeted drugs and treatment techniques. Gradient rotating magnetic field (RMF) is a new technology used in oncology physiotherapy, showing promising clinical applications due to its satisfactory biosafety and the abundant mechanical force stimuli it provides. However, its antitumor effects and underlying molecular mechanisms are not yet clear. We designed two sets of gradient RMF devices for cell culture and animal handling. Gradient RMF exposure had a notable impact on the F-actin arrangement of MDA-MB-231, BT-549, and MDA-MB-468 cells, inhibiting cell migration and invasion. A potential cytoskeleton F-actin-associated gene, CCDC150, was found to be enriched in clinical TNBC tumors and cells. CCDC150 negatively correlated with the overall survival rate of TNBC patients. CCDC150 promoted TNBC migration and invasion via activation of the transforming growth factor β1 (TGF-β1)/SMAD3 signaling pathway in vitro and in vivo. CCDC150 was also identified as a magnetic field response gene, and it was marked down-regulated after gradient RMF exposure. CCDC150 silencing and gradient RMF exposure both suppressed TNBC tumor growth and liver metastasis. Therefore, gradient RMF exposure may be an effective TNBC treatment, and CCDC150 may emerge as a potential target for TNBC therapy.

## Introduction

Breast cancer is the most prevalent malignancy in women worldwide [1]. Triple-negative breast cancer (TNBC) is defined by the absence or extremely low expression of breast cancer-specific antigens, such as human epidermal growth factor receptor-2 (HER-2), estrogen receptor (ER), and progesterone receptor (PR) [2,3]. TNBC is clinically characterized by extreme aggressiveness, high metastatic potential, and a low survival rate [4,5]. Neoadjuvant chemotherapy and surgical resection are used for the clinical treatment of TNBC, but these approaches lead to tumor recurrence due to drug resistance [6]. The absence of effective therapeutic targets poses a great challenge in TNBC clinical treatment [7]. Therefore, screening for effective targets and exploring novel treatment techniques are critical for TNBC treatment.

Physiotherapy, such as electricity, magnetism, cold, laser, ultrasound, capacitance, radiofrequency ablation, microwave coagulation, and systemic thermotherapy, has emerged as a novel approach for tumor treatment [8–11]. Among these, magnetic field therapy has gained prominence as a noninvasive and safe treatment compared to other modalities. The therapeutic potential of magnetic field therapy in inhibiting tumor growth has been substantiated in numerous studies, and its use in malignancy treatment dates back to the last century [12–14]. Magnetic field therapy is categorized into static and alternating magnetic fields [15]. Most of these studies focused on static magnetic fields. Notably, static magnetic fields with limited characteristics produce vastly different biological effects due to variations in magnetic field induction, treatment, and sample size [16]. Devices with high-intensity, static magnetic fields are often bulky and may pose unforeseen biosafety risks, hindering their clinical application [17].

Compared to static magnetic field devices, alternating magnetic fields offer a broader range of characteristics, such as magnetic field induction, spatial magnetic field gradient, and frequency. Among the various alternating magnetic fields, the gradient rotating magnetic field (RMF) is particularly prominent, exhibiting considerable potential in clinical applications and tumor suppression. Nie et al. [18] found that the hepatoma tumor growth in nude mice was inhibited after gradient RMF exposure for 30 d. Zheng and Zhang [19] found that the total efficiency of 100 patients with advanced malignant tumors increased to 54% after continuous gradient RMF exposure for 2 months. However, the biological effects of gradient RMF treatment on TNBC and the underlying mechanisms require further extensive investigation. Current gradient RMF devices have some disadvantages, such as the risk of electromagnetic thermal damage and limited magnetic field characteristics. Therefore, it is crucial to develop gradient RMF devices with a more extensive range of magnetic field characteristics.

The cytoskeleton is crucial for maintaining cell morphology and providing internal organization support, exhibiting sensitivity to mechanical stimuli from the cellular environment. Realignment of microtubules under magnetic fields induces a variation in cell morphology [20]. McFarlane et al. [21] found that the polarization coefficient of tumor cells increased significantly after the application of alternating magnetic fields on PC12 cells for 96 h. Ashdown et al. [22] showed that the morphology of breast cancer cells was remarkably elongated, and the integrity of the cell membrane was disrupted, which led to the inhibition of tumor cell viability, after exposure to alternating pulsed magnetic fields. However, the relative molecular mechanism of gradient RMFs affecting the tumor cytoskeleton has not been elucidated, and further in-depth research is needed.

Coiled-coil domain-containing (CCDC) proteins constitute a class of homo-oligomeric or oligomeric sequence proteins containing two or more coiled-coil structural domains, crucial for the assembly and arrangement of the cytoskeleton [23]. CCDCs participate in the formation of backbone proteins and the polymerization of actin, which is pivotal in driving the epithelial–mesenchymal transition of tumor cells [24]. Abnormal up-regulation of some CCDCs is found in tumors, and they are positively correlated with tumor metastasis [25,26]. For instance, CCDC6 exhibits aberrantly high expression in thyroid cancer, and CCDC66 is significantly up-regulated in colorectal cancer to promote tumor invasion and metastasis [27]. We found that the expression of CCDC150 in TNBC was significantly increased, which was subsequently down-regulated after gradient RMF exposure, while further investigations are needed to clarify the relative molecular mechanism.

In this study, we designed two gradient RMF devices for cell culturing and animal handling. The F-actin-related gene CCDC150, which might respond to gradient RMF exposure, was identified. The effect of gradient RMF on TNBC progression and metastasis via the regulation of CCDC150 was investigated both in vitro and in vivo, and the relative molecular mechanism was examined. This study proposes gradient RMF as a novel and effective physical technology for TNBC.

## Results

### Effect of gradient RMFs on the morphology, cytoskeleton, migration, and invasion capacity of MDA-MB-231 cells

**Fig. 1.**
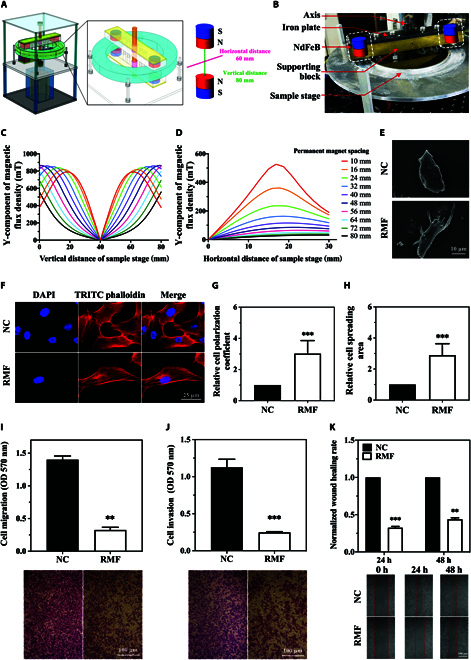
Gradient RMF cell culture device and its effect on cytoskeleton and motility of MDA-MB-231 cell at 5 Hz, 0.41 T. (A) Schematic diagram of gradient RMF cell culture platform. (B) Partial enlargement of gradient RMF device. Y-component of the magnetic flux density distribution in the vertical (C) and horizontal (D) directions in N–N mode. Effect of gradient RMF exposure on morphology (E), cytoskeleton (F), polarization coefficient (G), spreading area (H), migration (I), invasion (J), and wound-healing capacity (K) of MDA-MB-231 cells. *n* = 3. Statistical analysis was conducted using *t* test and one-way ANOVA. *P < 0.05, **P < 0.01, and ***P < 0.001 versus NC group.

The gradient RMF device used in this study was designed and processed in our laboratory [28], and the temperature control system was remodeled to adapt to cell culture requirements. The structure and partially enlarged drawing of this device are shown in Fig. 1A and B, respectively. A physical diagram of the gradient RMF cell culture device is shown in Fig. S1. The simulation results of ANSYS revealed that this combined magnetic field, with a strong horizontal direction, exhibited a more notable periodic variation in magnetic induction at the sample stage. Our previous study revealed that the gradient RMF device with an N–N pole arrangement induced significant aggregation and confinement effects on the magnetic material [28]. Furthermore, regarding the N–N pole, the horizontal magnetic field induction using the N–N pole at the sample stage was large, but the vertical magnetic fields canceled each other out (Fig. 1C). In the simulation of the horizontal magnetic induction intensity (Y-component) of the sample stage with different spacing arrangements between two N48M permanent magnets, the horizontal line at the sample stage was set to 60 mm, and the midpoint of the permanent magnet was set to 30 mm (considered the reference point for both magnets). The distribution of the magnetic induction intensity of the permanent magnets was analyzed, and the spacing was 60 mm horizontally at a distance ranging from 10 mm to 80 mm. The simulation results for the Y-component of the magnetic flux density in the horizontal direction are shown in Fig. 1D. The magnetic flux density at the midpoint of the magnet pairs gradually decreased with an increasing distance between the face-to-face NdFeB magnet pairs.

Magnetic field induction and frequency are the primary characteristics of the gradient RMF cell culture unit. Based on the magnetic field simulation results, the gradient RMF cell culture device provides a magnetic field induction range of 0 to 0.6 T in theory, and it is modulated by adjusting the spacing between magnets. However, the length of the device spindle limits the maximum space between the magnets, and the height of the sample tray limits the minimal space. Therefore, the magnetic space is only adjustable within a certain range, which results in providing a magnetic field induction within 0.04 to 0.41 T. The maximum frequency of the device is determined by the maximum power of the motor, the vibration, and heat generated during operation. The maximum frequency of this device is 5 Hz. Some studies used an electromagnetic field to achieve a higher frequency, but the exposure time was short due to heat and other issues. Therefore, we set different magnetic field induction (0.04, 0.2, and 0.41 T) and frequencies (2.5 and 5 Hz) for further investigation.

The optimal rotational frequency and magnetic field strength were determined by their effects on cell function. To determine the optimal magnetic field frequency, we first fix the magnetic field induction as 0.41 T to compare the effects of two frequencies (2.5 and 5 Hz) on MDA-MB-231 cell function. Since various cell function experiments require different treatment durations, the gradient RMF exposure duration varied accordingly. The results showed that morphology of MDA-MB-231 cell was altered after gradient RMF exposure at 2.5 and 5 Hz (0.41 T, 72 h) (Fig. 1E and F and Fig. S2A and D), and also both cell spreading area ratio and polarization coefficient increased more significantly in 5 Hz (Fig. 1H and G) than in 2.5 Hz (Fig. S2B and C). The inhibitory effects on migration, invasion, and wound-healing capacity of MDA-MB-231 cells were more significant after 5-Hz exposure than after 2.5-Hz exposure (Fig. 1I to K and Fig. S2E to G). Therefore, 5 Hz was selected as the optimal frequency for subsequent investigations.

Subsequently, the effects of different magnetic field inductions (0.04, 0.2, and 0.41 T) on the MDA-MB-231 cellular functions were compared at a fixed frequency of 5 Hz. The results showed that the cytoskeleton (0.41 T, 5 Hz, 72 h), migration (0.41 T, 5 Hz, 24 h), invasion (0.41 T, 5 Hz, 24 h), and wound-healing (0.41 T, 5 Hz, 48 h) capacity of MDA-MB-231 cells were more significantly affected compared with 0.04- and 0.2-T gradient RMF exposure (Fig. 1 and Fig. S3), and the 0.41-T treatment group exhibited more substantial increases in polarization and spreading area of the cytoskeleton. Based on these findings, we selected 5 Hz and 0.41 T as the gradient RMF characteristics for all subsequent experiments.

To verify the inhibitory effect of gradient RMF optimal parameters on TNBC cells, it was further studied in other subtype TNBC cells. TNBC is highly heterogeneous and is classified into seven subtypes based on gene expression characteristics: TNBC basal-like 1 (BL1), basal-like 2 (BL2), immunomodulatory (IM), mesenchymal (M), mesenchymal stem-like (MSL), luminal androgen receptor (LAR), and unspecified subtype (UNS). Therefore, we also selected MDA-MB-231 cells (MSL subtype), BT549 cells (M subtype), and MDA-MB-468 cells (BL1 subtype) for further testing. The effects of optimal gradient RMF parameters on the morphology, cytoskeleton, migration, and invasion capacity of these three TNBC cell lines (MDA-MB-231, BT549, and MDA-MB-468) were investigated. Notably, the morphology of MDA-MB-231 cells changed significantly under gradient RMF exposure, as confirmed by scanning electron microscopy (SEM). Gradient RMF exposure (0.41 T, 5 Hz, 72 h) significantly deformed the cytoskeleton F-actin (Fig. 1F) and led to increase in the polarization coefficient (Fig. 1G) and spreading area (Fig. 1H) compared to the control. F-actin underwent noticeable rearrangement, with a tendency for parallel alignment. Similar cytoskeletal deformations and polarization were also observed in BT549 and MDA-MB-468 cells (Figs. S4 and S5). The migration, invasion, and wound-healing capacity of MDA-MB-231 cells were significantly reduced after gradient RMF exposure (0.41 T, 5 Hz) (Fig. 1I to K), and similar effects were observed in BT549 and MDA-MB-468 cells (Figs. S4 and S5). Therefore, we hypothesized that cell behavior was modified under gradient RMF exposure through the rearrangement of cytoskeletal F-actin.

### Screening of the key gradient RMF-responsive cytoskeleton-related gene CCDC150 in TNBC

**Fig. 2.**
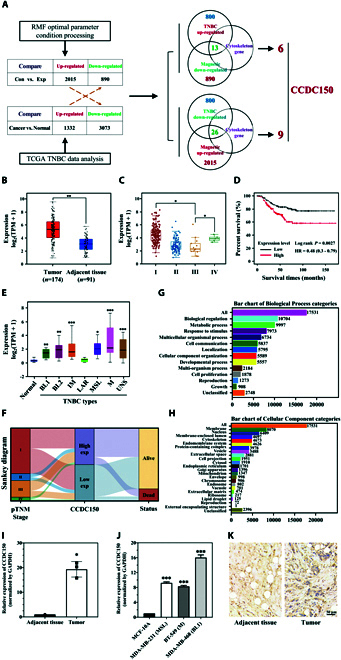
Correlation between CCDC150 expression and TNBC. (A) Screening of key gradient RMF response cytoskeleton-related gene CCDC150 in TNBC. (B) CCDC150 expression in TNBC tumor and adjacent tissue in TCGA database. (C) Correlation between CCDC150 expression and stage of tumor development according to TCGA database. (D) Effect of CCDC150 expression on overall survival rate in TNBC. (E) Expression of CCDC150 in different subtypes of TNBC. (F) Sankey diagram of CCDC150 expression in TNBC. Biological process (G) and cellular component categories (H) of CCDC150 in TNBC. CCDC150 expression in TNBC tumor tissues of clinical patients (I) and TNBC cells (J). (K) Immunohistochemical staining results of CCDC150 expression in TNBC tumor tissues. *n* = 3. Statistical analysis was conducted using *t* test, one-way ANOVA, or two-way ANOVA, and post hoc tests were carried out. *P < 0.05, **P < 0.01, and ***P < 0.001 versus NC group.

We simultaneously screened gradient RMF-responsive and aberrantly regulated breast cancer genes. We analyzed the transcriptome of gradient RMF-exposed TNBC cells and found that 2,015 genes were up-regulated and 890 genes were down-regulated in TNBC cells after gradient RMF exposure treatment compared to the control. A total of 1,332 genes were up-regulated, and 3,073 were down-regulated in TNBC tumor tissues according to the The Cancer Genome Atlas (TCGA) database. We obtained a dataset of cytoskeleton-related genes via an extensive literature survey and online dataset analysis. CCDC150 was significantly up-regulated in TNBC tumors, and it was down-regulated after gradient RMF treatment. Intersection analysis of these three datasets revealed that CCDC150 may be a key gradient RMF response cytoskeleton-related gene in TNBC (Fig. 2A). In addition, proteins with a large number of helices exhibit enhanced responsiveness to magnetic fields [24,29–31]. The predicted spatial structure of CCDC150 by AlphaFold (https://alphafold.ebi.ac.uk/entry/Q8NCX0) indicates its enrichment with helices, and CCDC150 may be more susceptible to diamagnetic anisotropy. Therefore, we focused on CCDC150 in the present study.

Our analysis revealed a significant increase in CCDC150 expression, with a 5.32-fold (*P* < 0.01) up-regulation in TNBC patient tumors compared to adjacent tissues (Fig. 2B). CCDC150 expression increased significantly in stage I and stage IV tumor nodal metastasis (TNM) compared to stage III (Fig. 2C). Its high expression positively correlated with poor survival in TNBC patients (Fig. 2D). Furthermore, it was confirmed that CCDC150 was highly expressed in BL1, BL2, IM, MSL, M, and UNS subtypes (Fig. 2E). RNA-sequencing data and corresponding clinical information of TNBC patient tumors (https://portal.gdc.com) and a Sankey diagram of CCDC150 were constructed (Fig. 2F), and it confirmed that high expression of CCDC150 was linked to poor patient prognosis. Bioinformatics analysis showed that CCDC150 was closely related to cellular component organization (Fig. 2G) and the cytoskeleton (Fig. 2H). We detected CCDC150 expression in TNBC patients, and the results showed that CCDC150 was up-regulated 19.31-fold (*P* < 0.05) in TNBC tumors compared to adjacent tissues (Fig. 2I). We also assessed the expression levels of CCDC150 in TNBC cells, and CCDC150 was increased 9.23-fold (*P* < 0.001) in MDA-MB-231 cell, 7.68-fold (*P* < 0.001) in BT549 cell, and 16.04-fold (*P* < 0.001) in MDA-MB-468 cell compared to MCF10A cell (Fig. 2J). This result was verified using immunohistochemical staining with CCDC150-specific antibody (Fig. 2K). These results confirmed that CCDC150 expression positively correlated with TNBC occurrence.

### CCDC150 promotes the invasion, migration, and cytoskeleton F-actin rearrangement in TNBC cells

**Fig. 3.**
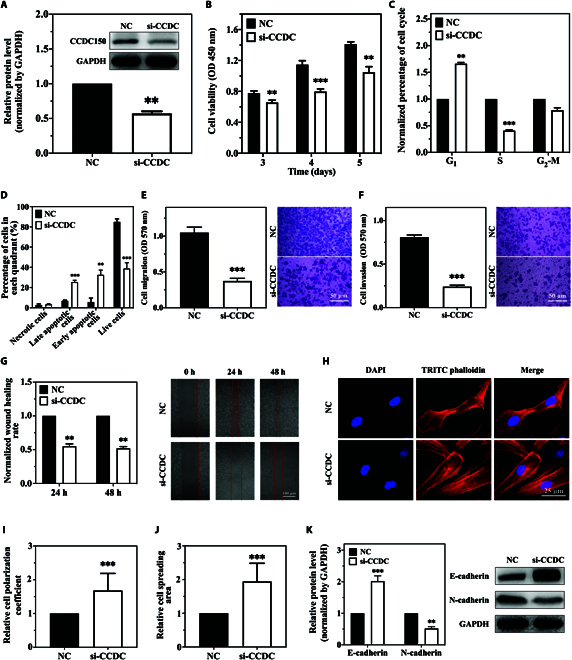
Effects of CCDC150 on migration, invasion, and cytoskeleton F-actin rearrangement in MDA-MB-231 cells. (A) Transfection efficiency after treatment with CCDC150-specific siRNA. Effect of CCDC150 knockdown on cell viability (B), cycle (C), apoptosis (D), migration capacity (E), invasion capacity (F), and wound-healing capacity (G) of MDA-MB-231 cells. Fluorescent staining of cytoskeleton F-actin in MDA-MB-231 cells (H), and cell polarization coefficient (I) and spreading area (J) after CCDC150 silencing. (K) Expression level of EMT biomarkers after CCDC150 silencing. GAPDH was used as a reference gene. *n* = 3. Statistical analyses were conducted using *t* test and one-way ANOVA. *P < 0.05, **P < 0.01, and ***P < 0.001 versus NC group.

To investigate the effect of CCDC150 on TNBC cell function, we designed a specific small interfering RNA (siRNA) (si-CCDC150) for transfection. CCDC150 expression was significantly down-regulated in MDA-MB-231 cells (Fig. 3A). The viability of MDA-MB-231 cells was reduced after CCDC150 knockdown (Fig. 3B), which may be due to massive cell arrest in the G_1_ phase (Fig. 3C). After transfection with si-CCDC150, the early and late apoptotic indices increased compared to the control, as detected using flow cytometry (Fig. S6). These results indicated a significant promotion of MDA-MB-231 cell apoptosis (Fig. 3D). The migration, invasion, and wound-healing rates of MDA-MB-231 cells were significantly reduced after CCDC150 knockdown (Fig. 3E to G). Similar trends were observed in BT549 and MDA-MB-468 cells after knockdown of CCDC150 (Figs. S7 and S8). The cytoskeletal F-actin of MDA-MB-231 cells was rearranged after CCDC150 knockdown (Fig. 3H), and its polarization coefficient and spreading area increased significantly (Fig. 3I and J). Similarly, it is observed that F-actin underwent rearrangement and exhibited a tendency to align in parallel. The results were similar in BT549 and MDA-MB-468 cells (Figs. S7 and S8). Therefore, MDA-MB-231 cells were used in subsequent experiments. We also examined the effect of CCDC150 on epithelial–mesenchymal transition (EMT) marker levels (Fig. 3K). There was a 2.04-fold (*P* < 0.001) increase in E-cadherin and a 48.13% (*P* < 0.01) decrease in N-cadherin after knockdown of CCDC150. The impact of the combination treatment with gradient RMF and si-CCDC150 was also investigated. The results showed that the migration, invasion, and wound-healing capacity of MDA-MB-231 cells were reduced in the combination treatment group compared to the single gradient RMF treatment group (Fig. S9A to C). The arrangement of F-actin was notably altered after treatment with gradient RMF + si-CCDC150 (Fig. S9D), which was accompanied by an increase in the polarization coefficient and cell spreading area (Fig. S9E and F). These findings demonstrated that CCDC150 silencing inhibited the metastatic capacity of TNBC cells and promoted F-actin rearrangement, and these effects were further enhanced via treatment combination with gradient RMF.

### Gradient RMF exposure or CCDC150 knockdown inhibits in vivo tumor growth

**Fig. 4.**
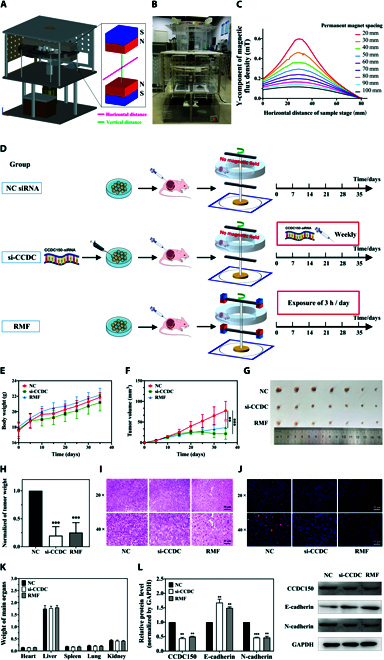
Effect of gradient RMF exposure or CCDC150 knockdown on TNBC tumor growth in situ. Schematic diagram (A) and profile display (B) of gradient RMF animal handling device. (C) Y-component of the magnetic flux density distribution with different spacing alignments in N–N mode. (D) Flow chart of experiments with gradient RMF exposure or CCDC150 knockdown of TNBC xenografts in nude mice. Body weight (E), tumor volume (F), tumor image (G), and tumor weight (H) in different groups (NC siRNA treatment, si-CCDC150 treatment, gradient RMF exposure). H&E (I) and Ki67 (J) staining of tumor tissue. (K) Major organ weights of mice in each group. (L) Expression level of CCDC150 and EMT biomarkers in different treatment groups. GAPDH was used as a reference gene. *n* = 7. Statistical analyses were conducted using *t* test, one-way ANOVA, or two-way ANOVA, and post hoc tests were carried out. *P < 0.05, **P < 0.01, and ***P < 0.001 versus NC group.

Because gradient RMF exposure and CCDC150 knockdown inhibited the migration and invasion capacity of MDA-MB-231 cells, their effects on TNBC tumor growth were validated in vivo. A noncontact gradient RMF animal handling device was designed based on the gradient RMF cell culture platform. Its stereo design and profile are shown in Fig. 4A and B, respectively. We set the center point between the two permanent magnets as the reference point. The horizontal line of the sample stage was 160 mm, and the midpoint of the permanent magnets was 80 mm. The Y-component of the magnetic induction strength in the range of 0 to 160 mm was subsequently analyzed, and the Y-component of the magnetic flux density was calculated using ANSYS (Fig. 4C). The gradient RMF animal handling device was established with an N–N pole arrangement and a magnetic field induction and frequency of 0.41 T and 1.33 Hz, respectively. We used the same magnetic induction and mode as the cell experiments but decreased the frequency in vivo to reduce the mechanical vibration of the device.

A total of 21 tumor-bearing nude mice were randomly divided into three groups: (a) NC siRNA without gradient RMF exposure, (b) si-CCDC150 treatment without gradient RMF exposure, and (c) gradient RMF exposure for 3 h per day. The experimental procedures are shown in Fig. 4D. All mice were sacrificed after 35 d of treatment. The body weight of mice continuously increased during treatment in all three groups, and the difference was not significant (Fig. 4E). Tumor volumes and weights decreased significantly in the si-CCDC150 and gradient RMF groups compared to the NC group. Tumor volumes decreased 68.1% (*P* < 0.001) and 59.93% (*P* < 0.01) after si-CCDC150 treatment and gradient RMF exposure, respectively (Fig. 4F and G). Tumor net weights decreased 80.21% (*P* < 0.001) and 74.83% (*P* < 0.001) in the si-CCDC150 treatment and gradient RMF exposure groups, respectively, compared to the NC group (Fig. 4H). Hematoxylin and eosin (H&E) staining revealed significant pathological changes in tumors after si-CCDC150 treatment or gradient RMF exposure (Fig. 4I). The nuclei of tumor cells were densely and uniformly arranged in the NC group. However, there was a significant reduction in the number of nuclei that exhibited a more dispersed pattern after si-CCDC150 treatment or gradient RMF exposure. Ki67 staining results further confirmed that the growth of tumor cells was significantly inhibited after gradient RMF exposure and si-CCDC150 treatment (Fig. 4J). There was no significant variation in the pathology or weight of any major organ (Fig. 4K and Fig. S10). CCDC150 protein expression levels decreased 56.12% (*P* < 0.01) and 50.01% (*P* < 0.01) in the si-CCDC150 treatment and gradient RMF exposure groups, respectively (Fig. 4L). si-CCDC150 treatment and gradient RMF exposure decreased N-cadherin levels and reversed the trend of E-cadherin levels (Fig. 4L). The combined treatment effect of gradient RMF and si-CCDC150 was also assessed. There was no significant difference in tumor weight between the gradient RMF treatment group and the gradient RMF treatment + si-CCDC150 group, and similar results were observed with H&E staining, Ki67, and liver function (Fig. S11A to H). There are two possible explanations: (a) The combination therapy may not provide significantly better outcomes than a single treatment in vivo. Reppingen et al. [32] found that the combination of the tyrosine kinase inhibitor cabozantinib and radiotherapy significantly decreased the proliferation and monoclonal formation ability of 4T1 cells in vitro, but the combination therapy did not inhibit breast tumor growth in homozygous mice. One possible reason is that some combination therapies may inadvertently activate additional tumor signaling pathways to potentially promote tumor growth [33]. (b) In vivo experiments are inherently more complex than in vitro experiments. In vivo experiments involve a multitude of additional factors, including the interaction between tumor cells and fibroblasts, the interaction between tumor cells and immune cells, and the presence of secretory proteins, pro-angiogenic factors, and extracellular vesicles.

### Gradient RMF exposure or CCDC150 knockdown inhibits hepatic colonization and liver metastasis of MDA-MB-231 cells

**Fig. 5.**
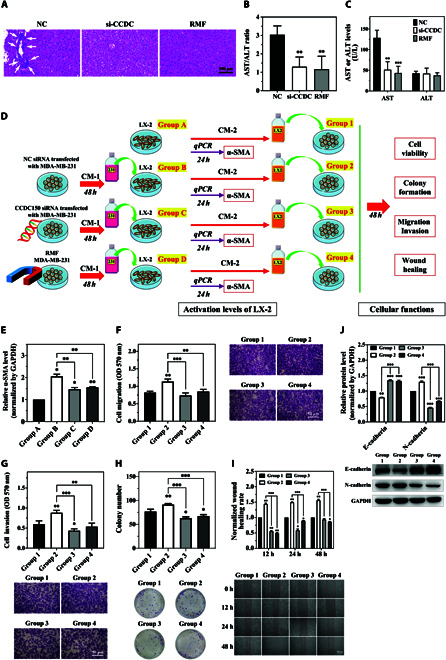
Gradient RMF exposure or CCDC150 silencing inhibits liver colonization and metastasis of MDA-MB-231 cells. (A) H&E staining images of liver tissues of nude mice. (B) ALT and AST level in nude mice. (C) ALT/AST ratio in nude mice. (D) Experiment flowchart of MDA-MB-231 cell coculture with LX-2 cells. (E) α-SMA levels in LX-2 cells after coculture with CM-1 collected from MDA-MB-231 cells (group A, normal medium; group B, CM-1 collected from MDA-MB-231 cells; group C, CM-1 collected from MDA-MB-231 cells after transfection with CCDC150 siRNA; group D, CM-1 collected from MDA-MB-231 cells after gradient RMF exposure). Effect of CM-2 from LX-2 cells on MDA-MB-231 cell migratory capacity (F), invasive capacity (G), colony formation capacity (H), wound-healing capacity (I), and EMT biomarker level (J). GAPDH was used as the reference gene. *n* = 3. Statistical analyses were conducted using *t* test, one-way ANOVA, or two-way ANOVA, and post hoc tests were carried out. *P < 0.05, **P < 0.01, and ***P < 0.001 versus NC group.

We isolated the major organs from tumor-bearing nude mice in different treatment groups and observed clear hepatic metastasis in the NC group but not in the gradient RMF exposure and si-CCDC150 treatment groups, which was confirmed using H&E staining (Fig. 5A). si-CCDC150 treatment and gradient RMF exposure inhibited the metastasis of MDA-MB-231 cells to liver in vivo. Glutamic oxaloacetic transaminase (AST) and glutamic pyruvic transaminase (ALT) levels indicate liver injury, and a high AST/ALT ratio may lead to liver cirrhosis, hepatitis, and liver cancer. The AST level and AST/ALT ratio decreased significantly after si-CCDC150 treatment and gradient RMF exposure (Fig. 5B and C). These results confirmed that liver injury was significantly alleviated after si-CCDC150 treatment or gradient RMF exposure.

The activation of hepatic stellate LX-2 cells is a key indicator of tumor cell colonization in the liver, especially the elevation of α-smooth muscle actin (α-SMA) expression. During the coculture of MDA-MB-231 and LX-2 cells, we divided LX-2 cells with different treatments into four groups: group A, normal medium; group B, conditional medium 1 (CM-1) collected from MDA-MB-231 cells; group C, CM-1 collected from MDA-MB-231 cells after transfection with CCDC150 siRNA; and group D, CM-1 collected from MDA-MB-231 cells after gradient RMF exposure (Fig. 5D). LX-2 cells in groups B, C, and D were cultured with CM-1 for 24 h, and normal culture medium was used for group A. The expression of α-SMA in different groups was detected, and the results revealed a significant decrease after si-CCDC150 treatment and gradient RMF exposure compared to NC (Fig. 5E). The composition of secretory proteins in conditioned media is highly complex, and these proteins have a significant impact on tumor growth and metastasis, such as the presence of certain chemokines [34,35].

To further investigate the effects of CCDC150 knockdown and gradient RMF exposure on TNBC liver metastasis, we used four treatment groups. The culture medium of LX-2 cells was collected in groups A, B, C, and D, which were designated conditional medium 2 (CM-2). In groups 1, 2, 3, and 4, MDA-MB-231 cells were cultured in CM-2 from groups A, B, C, and D, respectively (Fig. 5D). MDA-MB-231 cell migration, invasion, colony formation, and wound healing were significantly increased in group 2 compared to group 1, and they were decreased in groups 3 and 4 compared to group 2 (Fig. 5F to I). E-cadherin expression significantly increased after si-CCDC150 transfection or gradient RMF exposure compared to the control (Fig. 5J). These results collectively demonstrated that activation of LX-2 cells promoted TNBC hepatic colonization and metastasis, and these effects were reversed after CCDC150 silencing or gradient RMF exposure.

### Gradient RMF exposure inhibits TNBC progression by inactivating the CCDC150/TGF-β1/SMAD3 axis

**Fig. 6.**
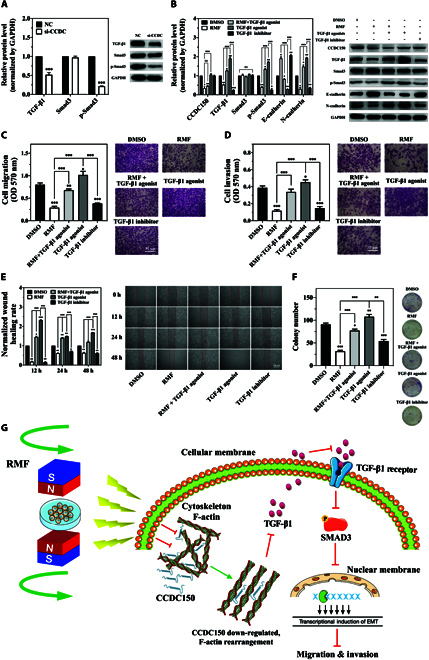
Gradient RMF exposure inactivates the TGF-β1/SMAD3 signaling pathway by down-regulating CCDC150 in MDA-MB-231 cells. (A) Expression of biomarkers of TGF-β1/SMAD3 signaling pathway after CCDC150 knockdown. (B) Expressions of TGF-β1/SMAD3 signaling pathway and EMT biomarkers after treatment with gradient RMF and TGF-β1 agonist and its inhibitor. Effect of gradient RMF exposure and TGF-β1/SMAD3 signaling pathway on migration (C), invasion (D), wound healing (E), and colony formation capacity (F) of MDA-MB-231 cells. (G) Potential mechanisms by which gradient RMF exposure suppresses TNBC progression by regulating CCDC150. GAPDH was used as the reference gene. *n* = 3. Statistical analyses were conducted using *t* test, one-way ANOVA, or two-way ANOVA, and post hoc tests were carried out. *P < 0.05, **P < 0.01, and ***P < 0.001 versus NC group.

To examine the potential molecular mechanisms underlying the inhibition of TNBC occurrence and progression after gradient RMF exposure and si-CCDC150 treatment, we screened downstream target genes that directly interact with CCDC150 using the cytoskeleton dataset and gene interaction analysis website. There were a significant correlation between CCDC150 and TGF-β1 expression in TNBC tumors (Fig. S12). Therefore, we hypothesized that TGF-β1 is a potential binding molecule. TGF-β1 was down-regulated by 48.13% (*P* < 0.001) after CCDC150 knockdown, and phosphorylated SMAD3 was significantly down-regulated by 78.67% (*P* < 0.001), but SMAD3 expression remained unchanged, which indicated that CCDC150 was essential for TGF-β1/SMAD3 activation (Fig. 6A). Moreover, compared with gradient RMF treatment, TGF-β1 and p-SMAD3 were further down-regulated when combined using si-CCDC150 (Fig. S9G).

We evaluated the effect of gradient RMF exposure on activation of the TGF-β1/SMAD3 pathway in five groups: (a) dimethyl sulfoxide (DMSO); (b) gradient RMF exposure; (c) gradient RMF + TGF-β1 agonist; (d) TGF-β1 agonist; and (e) TGF-β1 inhibitor groups. The half-maximal inhibition concentration (IC_50_) of SD208 and the half-maximal effective concentration (EC_50_) of SRI-011381 in MDA-MB-231 cells were 10 and 9.53 μM, respectively (Fig. S13A and B). As shown in Fig. 6B, CCDC150 expression was significantly down-regulated in the gradient RMF exposure and gradient RMF + TGF-β1 agonist groups compared to the DMSO group. In contrast, the CCDC150 level was rescued in the TGF-β1 agonist group, and it was significantly up-regulated compared to the gradient RMF exposure group. CCDC150 decreased after exposure to gradient RMF, and it simultaneously increased with the addition of the TGF-β1 agonist. However, treatment with TGF-β1 agonist or gradient RMF + TGF-β1 agonist did not rescue CCDC150 expression, which indicated that TGF-β1 was a downstream molecule of CCDC150. TGF-β1 was significantly down-regulated in the gradient RMF exposure group compared to the DMSO group, but it was up-regulated in the gradient RMF + TGF-β1 agonist and TGF-β1 agonist groups. In vitro treatment with the TGF-β1 agonist restored the protein expression of TGF-β1, and it was significantly up-regulated in the TGF-β1 agonist group compared to the gradient RMF exposure group. Phosphorylated SMAD and EMT markers showed a similar trend (Fig. 6B). These results suggested that the TGF-β1/SMAD3 signaling pathway was inactivated after exposure to gradient RMFs via the down-regulation of CCDC150, and it was activated by the TGF-β1 agonist. We evaluated the effect of the TGF-β1/SMAD3 signaling pathway on MDA-MB-231 cell viability (Fig. S14), migration, invasion, wound healing, and colony formation (Fig. 6C to F). They decreased significantly after exposure to gradient RMFs or treatment with TGF-β1 inhibitor, which was consistent with previous results. In contrast, they increased significantly after treatment with the TGF-β1 agonist or gradient RMF + TGF-β1 agonist (Fig. 6C to F). Collectively, these results demonstrated that gradient RMF exposure down-regulated the F-actin-related gene CCDC150, which inactivated the TGF-β1/SMAD3 axis to suppress TNBC progression and metastasis (Fig. 6G).

## Discussion

Magnetic field exposure has been gradually applied to various clinical applications, such as pain relief [36], anti-inflammatory and anti-swelling [37], tumor therapy [38], wound healing [39], nerve regeneration [40], osteonecrosis [41], and muscle function recovery [42]. Recent studies have shown that magnetic fields inhibited tumor growth in various cancers, such as breast cancer, neuroblastoma, fibroblastoma, liver cancer, cervical cancer, and colon cancer [43,44]. Although the relative mechanisms are not clear, in-depth mechanistic studies will contribute to its application in tumor therapy.

Various gradient RMF devices have been used in clinical applications. Zheng and Zhang [19] found a 54% increase in therapeutic efficacy in 100 patients with malignant tumors exposed to 0.4-T, 6.7-Hz gradient RMFs. Gradient RMF devices are typically classified into two main categories based on the magnets used, electromagnetic and permanent magnets. Thermal damage is always present in gradient RMF devices using electromagnetic magnets. Most gradient RMF devices are in the permanent magnet category [45]. However, the design and technical limitations of these devices result in a small frequency range, which ultimately limits their applications. In this study, two kinds of gradient RMF devices for in vitro and in vivo were designed and fabricated. The magnetic field inductions of gradient RMF cell culture and animal handling devices were 0.04 to 0.41 T (frequency: 0 to 5 Hz) and 0 to 1 T (frequency: 0 to 1.67 Hz), respectively. Compared to static magnetic field devices, these devices provide more abundant mechanical stimuli and characteristics to meet various experimental requirements.

The gradient RMF can provide higher values of magnetic force and stronger mechanical stimulation than uniform magnetic field. Numerous studies have found that exposure to gradient RMF significantly impeded the growth of tumor cells. Ren et al. [38] found that the proliferation of human lung adenocarcinoma A549 cells was inhibited, and apoptosis was promoted after continuous exposure to 0.4-T, 7.5-Hz gradient RMFs for 2 d. Zha et al. [46] found that treatment with gradient RMFs at 0.15 T, 4.2 Hz significantly inhibited tumor growth in breast cancer xenograft mice. In addition, Han et al. [47] found that gradient RMF treatment at 0.2 T, 4 Hz alleviated inflammatory deterioration and ameliorate immune dysfunction in ankylosing spondylitis model mice. The potential mechanisms of RMFs on cells can be summarized as follows: First, when cells are exposed to magnetic field, they can generate induced magnetic moment, exerting torque on intracellular components. The Lorentz force and magnetic moment generated by uniform magnetic field are proportional to induced strength of field. In the case of gradient magnetic field, the magnetic field gradient force is proportional to the square of gradient magnetic induction strength, resulting in a more robust Lorentz force [48]. Second, when cells are exposed to a magnetic field, various components within cell exhibit distinct magnetic field sensitivity and responsiveness [49,50]. After exposure to gradient magnetic field, the diverse constituents of cell may be influenced by the magnetic gradient force in different directions, ultimately leading to more intricate mechanical stresses within the cell. It is clear that mechanosensitive membrane ion channels can be affected by magnetomechanical stresses under gradient magnetic fields, with potassium ion channels being activated by stretching [51,52]. Additionally, Zablotskii et al. [53] revealed that the magnetic gradient force of gradient magnetic field can significantly affect the cytoskeleton and cell membrane according to the theoretical calculation. This was further validated by subsequent research [54]. Gradient RMF has exhibited positive effect on adjuvant therapy for malignant tumors and other diseases, while biological mechanism related to gradient RMF is still lacking. We performed an evaluation of gradient RMFs on TNBC cells, and the inhibitory effect of gradient RMFs on the cytoskeleton and metastatic ability was more pronounced with increasing magnetic field induction and frequency. Therefore, 5 Hz, 0.41 T was selected as the optimal characteristic for our gradient RMF cell culture device. Notably, gradient RMFs significantly inhibited the motility and invasive capacity of MDA-MB-231 cells, and these results were also verified in the M and BL1 subtype cells BT549 and MDA-MB-486. The BL1 and M subtypes are primarily composed of basal-like tumors, and the BL1 subtype is characterized by genomic instability due to a high rate of TP53 mutations and DNA repair-related gene deletions, which leads to a poor clinical prognosis. Gradient RMFs may offer promising new treatment options for these TNBC subtypes. We also found that gradient RMF exposure significantly inhibited tumor growth in TNBC tumor-bearing nude mice, which led to a substantial reduction in tumor volume and weight.

Cytoskeletons are fundamental to cell survival and activity. Tumor cells respond to the external environment and mediate a complex set of motor behaviors via their cytoskeletons. CCDC150 is a newly identified cytoskeleton assembly-related protein in the CCDC family, and it is involved in cytoskeleton assembly and actin polymerization [55]. Abnormal expression of CCDC in malignant tumors leads to cytoskeletal structural rearrangements, which activate intracellular signaling phospholipase C and mitogen-activated protein kinases to ultimately promote tumor cell migration and invasion [56]. CCDC150 was up-regulated in TNBC tumor tissues and cell lines, and its elevated expression was negatively correlated to poor clinical prognosis of TNBC patients. After CCDC150 knockdown in MDA-MB-231, BT549, and MDA-MB-468 cells, the polarization coefficient and spreading area of the cytoskeleton were significantly increased, with a substantial inhibition of cell invasion and tumor growth. The α-helix structure has a greater anisotropy due to the axial arrangement of the peptide bonds, and their overall diamagnetic anisotropy is greater when multiple bonds are arranged in a regular manner [31]. There are numerous helical structural domains in CCDC150, which enhance its diamagnetic anisotropy, and it may be the reason for CCDC150’s response to magnetic fields. Collectively, our results showed that CCDC150 acted as an oncogene that was suppressed via gradient RMF exposure, which highlights its potential as a target for drug design.

It was clear that gradient RMF inhibited TNBC progression, which led to substantial alterations in tumor cell morphology. However, the underlying physical mechanisms are not clear. The biological function of tumor cells is closely related to their morphology. Compared to normal cells, tumor cells undergo more obvious morphological changes, including morphological asymmetry, loss of cell polarity, damage to the cell membrane, and an increase in the nucleoplasmic ratio. These morphological changes promote the development of tumors and enhance the infiltration and metastasis of tumor cells. We confirmed that the polarity of TNBC cells increased after gradient RMF exposure, and the underlying physical mechanisms are worth discussing.

Cellular morphology is determined by internal stresses and external mechanical forces that act upon the cell membrane and cytoskeleton. The cell membrane plays a crucial role by maintaining a balance in its tension to safeguard the cytoskeleton against external mechanical forces. Conversely, the cytoskeleton provides internal structural support to static stress points and regulates mechanosensitive ion channels to protect the cell membrane. It is also involved in repairing the cell membrane following damage from external stress. Therefore, the cell membrane and cytoskeleton are critically important in driving changes in cell morphology. The effect of internal forces on cell morphology depends primarily on the cytoskeleton. F-actin is an important component of the cytoskeleton, which is sensitive to mechanical stimuli and plays an important role in the migration and invasion of tumor cells. Therefore, F-actin may be a target for magnetic treatment. Our study found that the polarization coefficient and the spreading area of cytoskeleton increased significantly after gradient RMF treatment. The impact of gradient RMF on the cytoskeletal distribution may be attributed to two primary factors: the disruption of F-actin arrangement and the application of compressive forces on the cell membrane.

First, the F-actin arrangement was disturbed under gradient RMF. Ji et al. [54] showed that F-actin formation was reduced after exposure to 0.4-T gradient RMFs and ultimately inhibited cell metastasis. Compared to their unilateral low-frequency gradient RMF, we used a bilateral N–N mode gradient RMF device. Considering the cell as a homogeneous two-dimensional magnetically induced material, the electrical potential induced by the bilateral gradient RMF device is significantly greater. The induced electromotive force *V* can be calculated according to Maxwell’s electromagnetic field theory, and *V* can be determined using the following equation:

$$V=-\frac{d\left(\mathrm{BS}\right)}{\mathrm{dt}}=-\pi \mathrm{Rr}\omega B$$ (1)

where *R* is the rotational radius of the permanent magnet, *r* is the radius of the cell sample, *ω* is the angular speed of rotation, and *B* is magnetic induction. When *R* and *r* are fixed, with the generation of gradient RMF-induced magnetic induction *B*, the induced electric potential increases with increasing *ω*. F-actin is a helical, filamentous macromolecule formed by the assembly of G-actin monomers. According to theoretical analysis by Ji et al. [54], the helical structure within F-actin undergoes significant electron hopping after exposure to a symmetrically distributed gradient RMF, which results in higher electrical conductivity and an increase in mechanical perturbation forces within F-actin.

Based on the G-actin monomer interaction energy analysis by Ji et al. [54], the thermal fluctuation energy generated within F-actin after gradient RMF exposure using our device was 1.05 × 10^−19^ J. Therefore, the mutual repulsion energy was 63 kJ/mol, which resulted in a repulsive force within F-actin of approximately 36 pN. Therefore, the gradient RMFs affect the charge redistribution between F-actin molecules and the interaction force. The conformation of F-actin is affected by the induced electric potential via coulombic forces, which affect cell morphology via stresses within the cytoskeleton to ultimately inhibit the metastatic ability of the tumor cells.

Second, gradient RMF is an alternating time-varying magnetic field, and the theoretical study of magnetically induced polarization demonstrated that cell membranes generated induced currents under alternating magnetic field stimulation, which ultimately resulted in cell membrane depolarization [57,58]. Induced currents produce magnetic stress by directly exerting magnetic pressure on the cell membrane [59], which leads to deformation of the cell membrane. In response, the cell membrane converts the mechanical signals induced by a magnetic field into a series of biological signals, which modulates various cellular functions, including gene expression, proliferation, apoptosis, migration, intracellular liquid–liquid phase separation, and extracellular matrix remodeling [60–62]. Alterations in cellular morphology result from intricate interactions between the cell membrane and the cytoskeleton, which collaborate to transmit the mechanical force signals induced by gradient RMF. We hypothesized that ions and magneto-sensitive molecules were redistributed after exposure to gradient RMFs. Therefore, the intracellular osmotic pressure changed due to the concentration difference between the intracellular and extracellular levels [63], and the cell membrane tension increased, which ultimately led to the activation or inactivation of corresponding signaling pathways. Gradient RMF treatment may induce changes in cell membrane tension and the conformation of F-actin via alterations in exogenous magnetic pressure and endogenous magnetic induction electromotive force. Therefore, cell morphology undergoes transformations as a result of the interactions between the cell membrane and the cytoskeleton. This transformation leads to inhibition of the metastatic and infiltrative capacity of tumor cells. Notably, these dynamics are only theoretical analyses. A more precise examination of the compression or stretching processes of tumor cells under the action of gradient RMF should be performed using accurate cell models and experimental methods.

TNBC tumors exhibit high aggressiveness and tissue-infiltrating characteristics, which lead to a high mortality rate. The liver and lung are its main distal metastatic organs [64]. We found liver metastases in TNBC tumors, but no metastases were detected in the gradient RMF exposure and CCDC150 siRNA treatment groups. To confirm the gradient RMF and CCDC150 silencing-mediated inhibition of hepatic metastasis, we established an in vitro coculture system of TNBC cells and hepatic astrocytes. LX-2 cells were activated after treatment with CM-1 from MDA-MB-231 cells. The migration, invasion, and colony formation abilities of MDA-MB-231 cells were significantly enhanced when these cells were cultured with CM-2 collected from LX-2 cells, and these results were reversed after gradient RMF exposure or CCDC150 siRNA treatment. These results indicated that gradient RMF exposure and CCDC150 silencing effectively prevented liver metastases in TNBC.

TGF-β1 is a member of the TGF-β family, and its heightened expression is associated with the enhancement of tumor invasion, migration, and immune evasion [65]. TGF-β1 is also closely related to chemoresistance in tumors, with elevated levels leading to resistance to chemotherapy and poorer prognoses [66]. The TGF-β1 signaling pathway plays an important role in the MSL subtype of TNBC. The MSL subtype is characterized by aberrant expression of genes associated with mesenchymalization of epithelial tissues. TGF-β1 is responsible for maintaining homeostasis in many epithelial tissues, and high expression of TGF-β1 in the MSL subtype is positively associated with an increased risk of axillary lymph node metastasis and a low patient survival rate [67]. Therefore, we investigated the effects of gradient RMF on the TGF-β1 signaling pathway in MDA-MB-231 cells of the MSL subtype. The TGF-β1/SMAD3 axis is a well-known signaling pathway associated with TNBC progression, and phosphorylation of SMAD3 promotes TNBC development and metastasis [68]. Therefore, we validated the correlation between CCDC150 and the TGF-β signaling pathway. Silencing of CCDC150 or gradient RMF exposure notably decreased TGF-β1 expression and SMAD3 phosphorylation levels. This reduction in TGF-β1 signaling impeded the migration and invasive capacity of MDA-MB-231 cells. However, TGF-β1 expression and SMAD3 phosphorylation levels recovered after treatment with a TGF-β1 agonist. These results confirmed that gradient RMF exposure inhibited TNBC progression by inactivating the TGF-β1/SMAD3 axis via the down-regulation of CCDC150. Numerous studies showed that TGF-β1 maintained the activation state of LX-2 cells and promoted hepatic fibrosis by inducing apoptosis and inhibiting hepatocyte proliferation, which induced the formation of the tumor microenvironment prior to liver metastasis [69]. We found an elevation in α-SMA levels after the introduction of a TGF-β1 agonist to LX-2 cells cultured with CM from MDA-MB-231 cells, which indicated that TGF-β1 was critical for the inhibition of liver metastasis after gradient RMF exposure or CCDC150 siRNA treatment. Collectively, our results indicated that gradient RMF exposure has great potential in the clinical treatment of TNBC in the future, and CCDC150 has emerged as a promising and new target for TNBC therapy.

## Conclusion

Breast cancer stands as the most prevalent malignancy among women worldwide, with TNBC exhibiting the highest recurrence and mortality rates. We designed gradient RMF cell culture and animal handling devices. Our results demonstrated that gradient RMF exposure inhibited cell migration, invasion, and tumor growth and affected the cytoskeleton of tumor cells. The possible underlying physical mechanisms of gradient RMF inhibition of the metastatic capacity of TNBC cells may occur through the reorganization of cytoskeletal F-actin. We identified a key cytoskeletal gene, CCDC150, which was abnormally elevated in TNBC tumor tissues and markedly down-regulated after gradient RMF exposure. CCDC150 knockdown effectively restrained TNBC cell metastasis and EMT via activation of the TGF-β1/SMAD3 axis. Gradient RMF exposure significantly inhibited tumor growth and distal liver metastasis in TNBC tumor-bearing nude mice by inactivating the TGF-β1/SMAD3 signaling pathway through the suppression of CCDC150 expression. Therefore, gradient RMF exposure is a novel and promising technology for TNBC treatment, with CCDC150 presenting itself as a potential target in this regard.

## Materials and Methods

### Gradient RMF cell culture device

The gradient RMF cell culture platform comprises several key components to ensure cell cultivation, including RMF cell culture unit, control cell culture unit, temperature control system, sterilization and clean system, and drive speed control system.

(a) RMF cell culture unit: It comprises two pairs of NdFeB cylinders (NNF48M; 40 mm diameter × 30 mm height) (SiQiang Technology, Shaanxi, China). These cylinders are securely affixed at both ends of parallel copper support rods (300 mm long × 50 mm wide × 30 mm thick). The NdFeB cylinder pair is assembled face-to-face. Each support rod is connected to a Q235 iron plate (350 mm long × 55 mm wide × 10 mm thick) to conduct the magnetic field. The upper and lower NNF48M pole orientations are set to N–N mode. The magnetic field induction can be enhanced by reducing the distance between the sets of NdFeB, which can be adjusted to a maximum distance of 60 mm. To ensure the structural integrity and parallel alignment of the support rods, four copper support shafts are employed. All the components are assembled and rotated around the central Q235 iron rod.

(b) Control cell culture unit: The only distinction in the control cell culture unit lies in the use of unmagnetized NdFeB cylinders instead of magnetized ones, as used in the RMF cell culture unit. The RMF cell culture unit and control cell culture unit are connected by a rotating shaft, enabling them to rotate simultaneously. They are separated by iron shielding plate to obstruct the magnetic field generated by the RMF cell culture unit, which was further confirmed by a Gaussmeter of type 421 (Lake Shore Cryotronics, Ohio, USA).

(c) Cell culture environment: The gradient RMF generation unit is embedded inside the incubator, and foam adhesive is used to seal the filter hole.

(d) Temperature control system: Eight water-cooled plate-type condensers, arranged in series around the motor, are interconnected with a water bath apparatus.

(e) Sterilization and cooling system: Ultraviolet (UV) lamps are used to regularly automatically sterilize the device. Two cooling fans are positioned at the top and bottom of the unit to facilitate gas circulation.

(f) Drive speed control system: The speed of the gradient RMF device ranges from 0 to 7.5 Hz. The physical diagram of gradient RMF cell culture device is shown in Fig. S1.

### Gradient RMF device for animal handling

The gradient RMF animal handling device is composed of a magnetic field generation unit, a magnetic rotating support unit, a power drive unit, and an animal tray. The magnetic field generation unit is composed of two pairs of NdFeB cubes (height, 50 mm; length, 100 mm; width, 100 mm) (NNF52M; SiQiang Technology, Shaanxi, China), which are fixed at the ends of stainless steel parallel support bars (700 mm long × 160 mm wide × 70 mm thick). Each NdFeB cube pair is assembled face-to-face, with the upper and lower opposing NNF52M pole orientations set to N–N mode. The structure of the animal handling device is similar to the cell device, allowing a maximum adjustment distance between magnets of 80 mm. Each magnet rotating support unit set has a magnet support arm base plate, and all the components rotate around the central stainless steel rod (diameter: 70 mm).

### Gradient RMF simulation

COMSOL Multiphysics 5.5 (COMSOL, Gothenburg, Sweden) was utilized to simulate the gradient RMF. The distribution and induction of the gradient RMF were analyzed using the three-dimensional static magnetic scalar analysis method.

The computational model of gradient RMF arrangement structure was established using ANSYS 19.2 (ANSYS, Pennsylvania, USA) finite element simulation. Subsequently, the magnetic induction variation curve was conducted by meticulously defining the gradient spacing between the permanent magnets, as outlined below:

(a) Modeling and parameterization: The actual size and arrangement of the permanent magnets of the gradient RMF devices were modeled. Parameters such as magnet remanence, magnetic permeability, magnetic coercive force, magnetic pole direction, distance between two magnets, and relative air permeability were set.

(b) ANSYS simulation: The air permeability was set to 1, and the model was meshed using the free mesh division mode. The relative permeability was selected as the magnetization model, which, in turn, was set as the residual flux density.

### Temperature and vibration stability testing of gradient RMF device

Thermocouples (KP1000; CHINO, Tokyo, Japan) were used to measure the temperature of the gradient RMF cell and the animal device. The thermocouple was connected to the sample stage, and the speed of the gradient RMF cell device was set to 5 Hz (1.3 Hz for the animal device). Temperature readings were recorded every 10 min over a 1-h period after a 20-min stabilization.

A laser displacement sensor (BL-30NZ; BOJKE, Shenzhen, China) was utilized to monitor amplitude changes in both the gradient RMF cell and the animal stage during operation. The sensor was positioned directly above the sample stage and detected amplitude variations by measuring the displacement between the laser sensor and the tray. Throughout the temperature stability testing, the frequency of device remained constant, and the amplitude was recorded every 0.1 s over 1 h. The results demonstrated that there was no significant heat generation or vibration in this study (Fig. S15).

### Cell lines and reagents

Human TNBC cells (MDA-MB-231, BT549, and MDA-MB-468), human normal mammary epithelial cells (MCF-10A), and human hepatic stellate cells (LX-2) were obtained from the National Model and Characterization Experimental Cell Bank (https://www.cellbank.org.cn). All the cells were cultured at 37 °C and maintained at 5% CO_2_, while no additional CO_2_ is supplemented for MDA-MB-231 cell. The culture medium of MDA-MB-231 cell is Leibovitz’s 15 medium (Servicebio, Wuhan, China), RPMI 1640 medium was for BT549 cell (Servicebio), and Dulbecco’s modified Eagle’s medium (DMEM) (Servicebio) was for the remaining cells. All the cell culture medium was supplemented with 10% fetal bovine serum (Vivacell, Shanghai, China) and 1% penicillin-streptomycin (Biological Industries, Beit-Haemek, Israel). The TGF-β1-specific inhibitor SD208 (Beyotime, Shanghai, China) and its specific activator SRI-011381 (MCE, Sovizzo Vicenza, Italy) were dissolved to 20 and 10 mM as stock by using DMSO (Solarbio, Beijing, China), respectively.

### Tumor tissues

All clinical tissue samples in this study, including tumor and paracancerous tissues from TNBC patients, were provided by the Department of Breast Surgery, First Hospital of Jilin University. None of the patient received chemotherapy or radiation therapy, and the tissues were frozen at −80 °C immediately after surgery. Informed consents were obtained from all patients for the use of their tissue samples in research.

### TCGA and tumor cytoskeleton data analysis

The comprehensive clinical database encompassing all breast cancer cases was obtained from the TCGA database (https://cancergenome.nih.gov/abouttcga/overview) using the Sangerbox data analysis platform (http://sangerbox.com/). Data analysis was performed using the R language toolkit and Sangerbox platform. The tumor cytoskeleton gene dataset was obtained from database screening (http://biogps.org/dataset/tag/cytoskeleton/, https://CySPID.com). Differentially expressed genes were identified based on a *P* value threshold ≤0.05 and a fold change ≥2.0. Key genes were screened out through the subsequent intersection of the two datasets.

### Cell transfection

CCDC150 siRNAs were designed by siRNA Gene Silencing (https://www.origene.com), and BLAST analysis was further used to confirm its specificity. The siRNA sequences are listed in Table S1. Both CCDC150 siRNAs and silencer negative control siRNAs were chemically synthesized by GenePharma (Shanghai, China) without further purification. Cells (0.5 × 10^6^ to 1 × 10^6^) were seeded on six-well plates for attachment, and 150 pmol of siRNA was transfected using 7.5 μl of GP-transfect-Mate according to the product instruction (GenePharma).

### Quantitative real-time PCR

Cells were collected after exposure to gradient RMFs for 72 h or transfection with siRNA for 24 h. Total RNA was extracted using TRIzol (Vazyme, Nanjing, China), and reverse transcription was carried out using Script RT Kit (Servicebio). Quantitative real-time polymerase chain reaction (qRT-PCR) was performed using the SYBR Green qPCR Kit (Servicebio). Glyceraldehyde-3-phosphate dehydrogenase (GAPDH) was used as a reference gene. All primers were chemically synthesized by Qingke (Beijing, China), and their sequences are shown in Table S2.

### Western blot analysis

After 72 h of gradient RMF exposure or 24 h of siRNA transfection, total protein was extracted from cell and tissue by using radioimmunoprecipitation assay (RIPA) solution with supplement of 1% phenylmethylsulfonyl fluoride (PMSF) (Solarbio), and then it was quantified by bicinchoninic acid assay (BCA) kit (Servicebio). Sodium dodecyl sulfate–polyacrylamide gels (6 to 12%) were prepared to separate protein, protein bands were transferred to polyvinylidene difluoride (PVDF) membranes (Millipore, Massachusetts, USA) and blocked with 5% skimmed milk (Yili Industrial Group Company, Inner Mongolia, China), and then membranes were incubated with primary antibody diluted solution for 12 h at 4 °C. The primary antibodies included anti-CCDC150 (1:600 dilution) from CUSABIO (Wuhan, China), 1:1000 dilution for TGF-β1 (ABclonal, Wuhan, China), 1:800 dilution for STAT3 (ABclonal), 1:900 dilution for p-STAT3 (ABclonal), 1:1200 dilution for E-cadherin (ABclonal), 1:1200 dilution for N-cadherin (ABclonal), and 1:1000 dilution for GAPDH (ABclonal). The membranes were then incubated with the secondary antibody at 1:4000 dilution (Zhuangzhi, Shaanxi, China) for 2 h at room temperature. Protein signals were detected using chemiluminescence solution (BOSTER, Wuhan, China), scanned by automated chemiluminescence imaging workstations (Tanon 6600, Shanghai, China), and then quantified using Image Lab 4.0 (National Institutes of Health, Maryland, USA).

### Cell viability assay

Cells (1 × 10^3^ per well) were seeded on 96-well plates. Cell Counting Kit-8 (CCK8) solution (10 μl per well) (Biosharp, Anhui, China) was added at 1, 2, 3, 4, and 5 d, and then they were incubated at 37 °C for 3 h. Absorbance was detected by using the Gen5 Microplate Reader (Biotek, Vermont, USA) at 450 nm, and the reference wavelength was 630 nm.

### Wound-healing assay

Cells (1 × 10^5^ per well) were seeded in 35-mm culture dishes. When cells reached 95% confluence, three straight lines were scratched perpendicular to the bottom of the culture dish using a 200-μl tip, and then they were photographed using a microscope at 0, 12, 24, and 48 h. The wound-healing area was quantified using ImageJ software (National Institutes of Health, Maryland, USA), and it was calculated as follows: cell migration area = area of the scratch (0 h) − area between scratches (12, 24, or 48 h). Scratch healing rate = cell migration area (12, 24, or 48 h)/scratch area (0 h).

### Transwell migration and invasion assay

A sterile phosphate-buffered saline (PBS) solution was used to dilute REF Matrigel (Corning, New York, USA) to 300 μg/ml, and then 100 μl of the matrix gel was added to the upper surface of transwell chambers. Cells (2 × 10^4^ per well), suspended in serum-free medium, were added to a transwell chamber without or with REF Matrigel for cell migration and invasion assays, respectively. Cells were fixed with 900 μl of 4% paraformaldehyde and stained with 1% crystal violet (Servicebio) after 24-h culture. Subsequently, the cells were washed with 33% precooled acetic acid and measured at 570 nm using a Biotek Epoch Full Wavelength Enzyme Labeler (Biotek, Vermont, USA).

### Colony formation assay

MDA-MB-231 cells (7 × 10^2^ per dish) were seeded in 35-mm cell culture dish. The culture medium was discarded when approximately more than 50 cells were present in each clone. Thereafter, 1 ml of 4% paraformaldehyde was added to fix cells, and 1% crystalline violet solution was used to stain the cells, then the images were photographed, and the number of monoclonal clones was counted.

### Cytoskeleton fluorescence staining

Cells (4 × 10^3^) were seeded on a sterile coverslip and fixed with a 4% formaldehyde solution. Tetramethyl rhodamine isothiocyanate (TRITC)-labeled ghost cyclic peptide solution (100 nM) and 4′,6-diamidino-2-phenylindole (DAPI) solution (100 nM) were used to stain the cytoskeleton and nuclei, respectively, after which the coverslips were dripped with an anti-quenching fluorescent blocker. Cell images were captured under a fluorescence microscope (Axio Observer 3; ZEISS, Baden-Württemberg, Germany) using 540/570 nm for TRITC and 364/454 nm for DAPI. In the gradient RMF exposure group, the cells were exposed to 5-Hz gradient RMFs for 72 h.

### Cell morphology detection

MDA-MB-231 cells (5 × 10^4^) were seeded on sterile coverslip and cultured for 48 h. Prechilled glutaraldehyde solution (4%) was added for 4 h, after which cells were dehydrated with gradient increase concentrations of ethanol (30 to 100%). Thereafter, the cells were dried for 2 h and subjected to gold spraying for 50 s. Cell morphology was captured under SEM (VEGA 3 SBH; TESCAN, Brno, Czech Republic); the accelerating voltage was 15 kV, and the working distance was 10 mm.

### Coculture of MDA-MB-231 and LX-2 cells

To detect the effect of MDA-MB-231 cells on the activation of LX-2 cells, we collected CM-1, which was used to culture MDA-MB-231 cells for 2 d, after which LX-2 cells were cultured with CM-1 for 1 d. To further evaluate the effect of CCDC150 and gradient RMF exposure on breast cancer liver metastasis, CM-2 was collected after culturing LX-2 cells for 2 d, which was then used to culture MDA-MB-231 cells to assess its biofunction.

### Xenograft model

All animal experiment protocols have been approved by Institutional Animal Care and Ethical Committee of the Animal Center of Northwestern Polytechnical University. Five- to 6-week-old female BALB/c nude mice were from Vital River (Beijing, China). Matrix gel was diluted to 5 mg/ml using precooled DMEM, which was free of fetal bovine serum and antibodies. NC siRNA or CCDC150 siRNA (600 pmol) was transfected into 2.5 × 10^6^ MDA-MB-231 cells for 24 h, and cells were resuspended by adding precooled matrix gel to 100 μl, and they were injected into the second pair of breast pads of each nude mouse. In addition, 10 μg of NC siRNA or CCDC150 siRNA was re-injected into each mouse once a week to prevent RNA degradation. The gradient RMF induction and frequency were set at 0.41 T and 1.5 Hz, respectively. All mice were exposed to gradient RMFs for 3 h/d (the exposure time was fixed between 9:00 and 12:00 a.m.) [18,38,70]. The body weight of nude mice was recorded daily using an electronic analytical balance (AUX320; Shimadzu, Kyoto, Japan). The long and short diameters of the tumor were measured daily using an electronic vernier caliper (Nscing Es, Nanjing, China). Tumor volumes were calculated according to the tumor volume formula and tumor growth curves: *V* = 0.5236 × *D*_1_ × (*D*_2_)^2^, where *D*_1_ and *D*_2_ are the long and short tumor diameter, respectively. All mice were sacrificed after 35 d, after which tumors, vital organs, and serum were collected.

### H&E, Ki67 immunofluorescence, and immunohistochemical staining

All tissue samples were fixed in 4% paraformaldehyde solution for 12 h, and tissue samples were embedded using paraffin wax. Hematoxylin kit (Servicebio) and Eosin staining kit (Servicebio) were used for staining, followed by sealing with neutral gum (Servicebio). For Ki67 staining, closure was performed using 5% bovine serum albumin (Servicebio). The samples were then incubated with anti-Ki67 polyclonal antibody (1:800 dilution) (Servicebio) and fluorescently labeled secondary antibody (1:2000 dilution) (Zhuangzhi, Shaanxi, China), and captured by fluorescence microscopy. Immunohistochemical staining was performed using tumor tissue sections from TNBC patients. The sections were permeabilized and blocked with 3% hydrogen peroxide, and then incubated with CCDC150 primary antibody (CUSABIO, Wuhan, China). The images were captured by fluorescence microscopy.

### Liver function analysis

Sera were collected from nude mice through eyeball extirpation, and liver function-related indicators were analyzed using a biochemical analyzer (Beckman Coulter, California, USA).

### Statistical analysis

The raw experimental data were analyzed using the SPSS statistical package version 24.0 (Armonk, New York, USA). Data are presented as mean ± standard deviation (SD), and each experiment was conducted at least three times for validation. In addition, *t* test, one-way analysis of variance (ANOVA), and two-way ANOVA were used to analyze data, and post hoc tests were also conducted. GraphPad Prism 9.0 software (GraphPad Software, California, USA) was used for statistical plotting. The overall survival rate was determined through Kaplan–Meier analysis. *P* < 0.05 indicated statistical significance.

## Data Availability

All data supporting the findings of this study are available within the paper and its Supplementary Materials. And the datasets generated or analyzed during this study are available from the corresponding author on reasonable request.
