# Supplementary material for: Gradient Rotating Magnetic Fields Impairing F-Actin-Related Gene CCDC150 to Inhibit Triple-Negative Breast Cancer Metastasis by Inactivating TGF-β1/SMAD3 Signaling Pathway
Source: Research (Wash D C). 2024 Feb 28;7:0320. doi: 10.34133/research.0320 (PMC10900498; doi:10.34133/research.0320)
Supplement: Supplementary 1 — Figs. S1 to S15 Tables S1 and S2 [file research.0320.f1.zip › Supplementary Data.pdf]

Supplementary Data

Gradient rotating magnetic fields impairing F-actin related gene CCDC150 to inhibit triple-negative breast cancer metastasis by inactivating TGF-β1/SMAD3 signaling pathway

Ge Zhang[1], Tongyao Yu[1], Xiaoxia Chai, Shilong Zhang, Jie Liu, Yan Zhou, Dachuan Yin [*], Chenyan Zhang [*]

Institute for Special Environmental Biophysics, Key Laboratory for Space Bioscience and Biotechnology, School of Life Sciences, Northwestern Polytechnical University, 710072, Xi'an, China

---

* Corresponding author. Institute for Special Environmental Biophysics, Key Laboratory for Space Bioscience and Biotechnology, School of Life Sciences, Northwestern Polytechnical University, Xi'an, 710072, China.

* *E-mail addresses:* yindc@nwpu.edu.cn (D.C. Yin), zhangchenyan@nwpu.edu.cn (C.Y. Zhang).

[1] These authors contributed equally to this work.

Institute email: npuinfo@nwpu.edu.cn

Table of Contents

**Supplementary Tables**		
S1	**Table S1.** siRNA sequences	P1
S2	**Table S2.** Primer sequences of qRT-PCR	P2
**Supplementary Figures**		
S1	**Figure S1.** Physical view of gradient RMF cell culture device	P3
S2	**Figure S2.** The temperature and vibration evolution in both of gradient RMF cell culture and animal feed platform	P4
S3	**Figure S3.** Effect of gradient RMF on F-actin, migration, invasion, and wound healing capacity of MDA-MB-231 cells at 2.5 Hz	P5
S4	**Figure S4.** Effect of gradient RMF on F-actin, migration, invasion, and wound healing capacity of MDA-MB-231 cells at different inductions of magnetic field	P7
S5	**Figure S5.** Effect of gradient RMF on F-actin, migration, invasion, and wound healing capacity of BT549 cells at optimal characteristics	P8
S6	**Figure S6.** Effect of gradient RMF on the F-actin, migration, invasion, and wound healing capacity of MDA-MB-468 cells at optimal characteristics	P9
S7	**Figure S7.** Effects of CCDC150 knocking-down on MDA-MB-231 cell apoptosis	P10
S8	**Figure S8.** Effect of CCDC150 on F-actin, migration, invasion, and wound healing capacity of BT549 cells	P11
S9	**Figure S9.** Effect of CCDC150 on F-actin, migration, invasion, and wound healing capacity of MDA-MB-468 cells	P12

S10	**Figure S10.** Effects of gradient RMF exposure or gradient RMF+si-CCDC150 combination treatment on MDA-MB-231 cells	P13
S11	**Figure S11.** Effects of gradient RMF exposure or si-CCDC150 on the major organs of TNBC-bearing nude mice	P15
S12	**Figure S12.** Effect of gradient RMF exposure or gradient RMF+siCCDC150 combination treatment on TNBC tumor growth *in situ*	P16
S13	**Figure S13.** ρSpearman correlation analysis of CCDC150 and TGF-β1	P17
S14	**Figure S14.** SD208 and SRI-011381 treatment concentration screening	P18
S15	**Figure S15.** Effect of gradient RMF and TGF-β1/SMAD3 signaling pathway on the MDA-MB-231 cell viability	P19

Supplementary Tables

Table S1. siRNA sequences

Gene	Sequence (5'-3')	
CCDC150 siRNA	Forward	GCAGGCCUCAAGAAAGAAATT
	Reverse	UUUCUUUCUUGAGGCCUGCTT

Table S2. Primer sequences of qRT-PCR

Gene name		Sequence (5'-3')
h-CCDC150	Forward	CTATTTGGAAGCTCCAGACTGTT
	Reverse	CGGCACATTCGATTTACCAGAA
E-cadherin	Forward	ACAGCCCCGCCTTATGATT
	Reverse	TCGGAACCGCTTCCTTCA
N-cadherin	Forward	AGAGGCCTATCCATGCTGAG
	Reverse	AGCAGCTTTAAGGCCCTCAT
h-TGF-β1	Forward	AAGGACCTCGGCTGGAAGTG
	Reverse	CCCGGGTTATGCTGGTTGTA
α-SMA	Forward	CGGAGCGCAAATACTCTGTC
	Reverse	TTTGCGGTGGACAATGGAAG
h-GAPDH	Forward	CTGACTTCAACAGCGACACC
	Reverse	TAGCCAAATTCGTTGTCATACC

Supplementary Figures

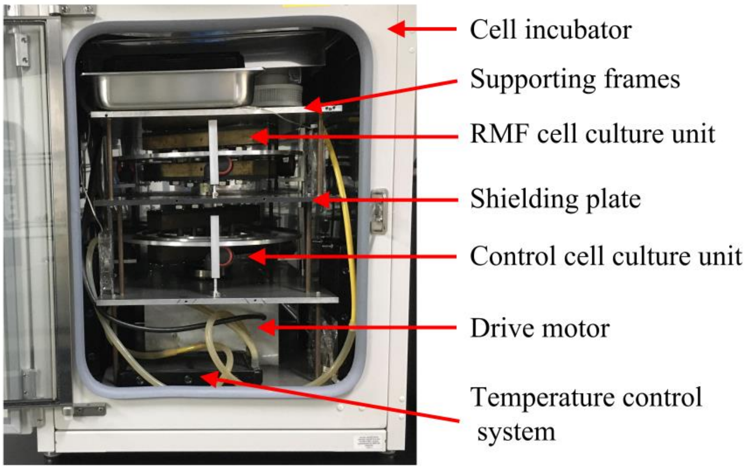

Figure S1. Physical view of gradient RMF cell culture device.

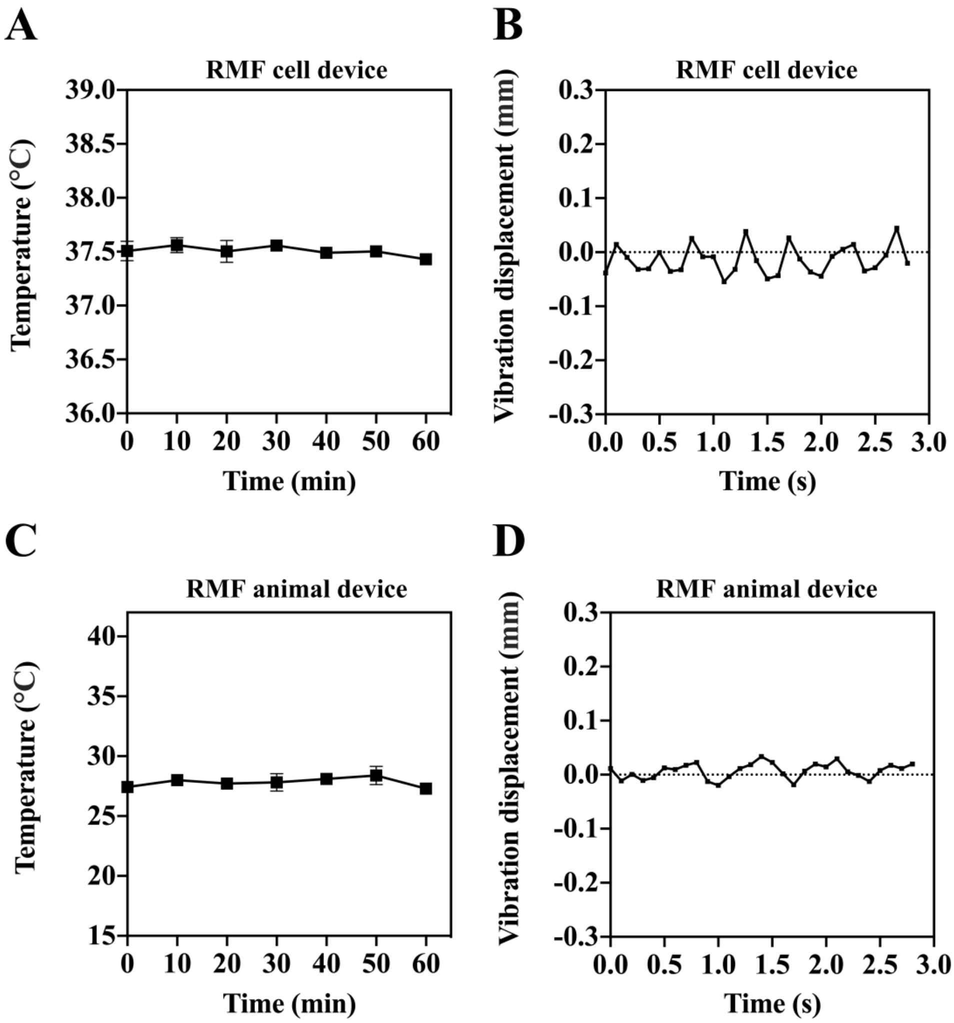

Figure S2. The temperature and vibration evolution in both of gradient RMF cell culture and animal feed platform. Temperature (A) and vibration (B) evolution in gradient RMF cell culture platform. Temperature (C) and vibration (D) evolution in gradient RMF animal feed platform.

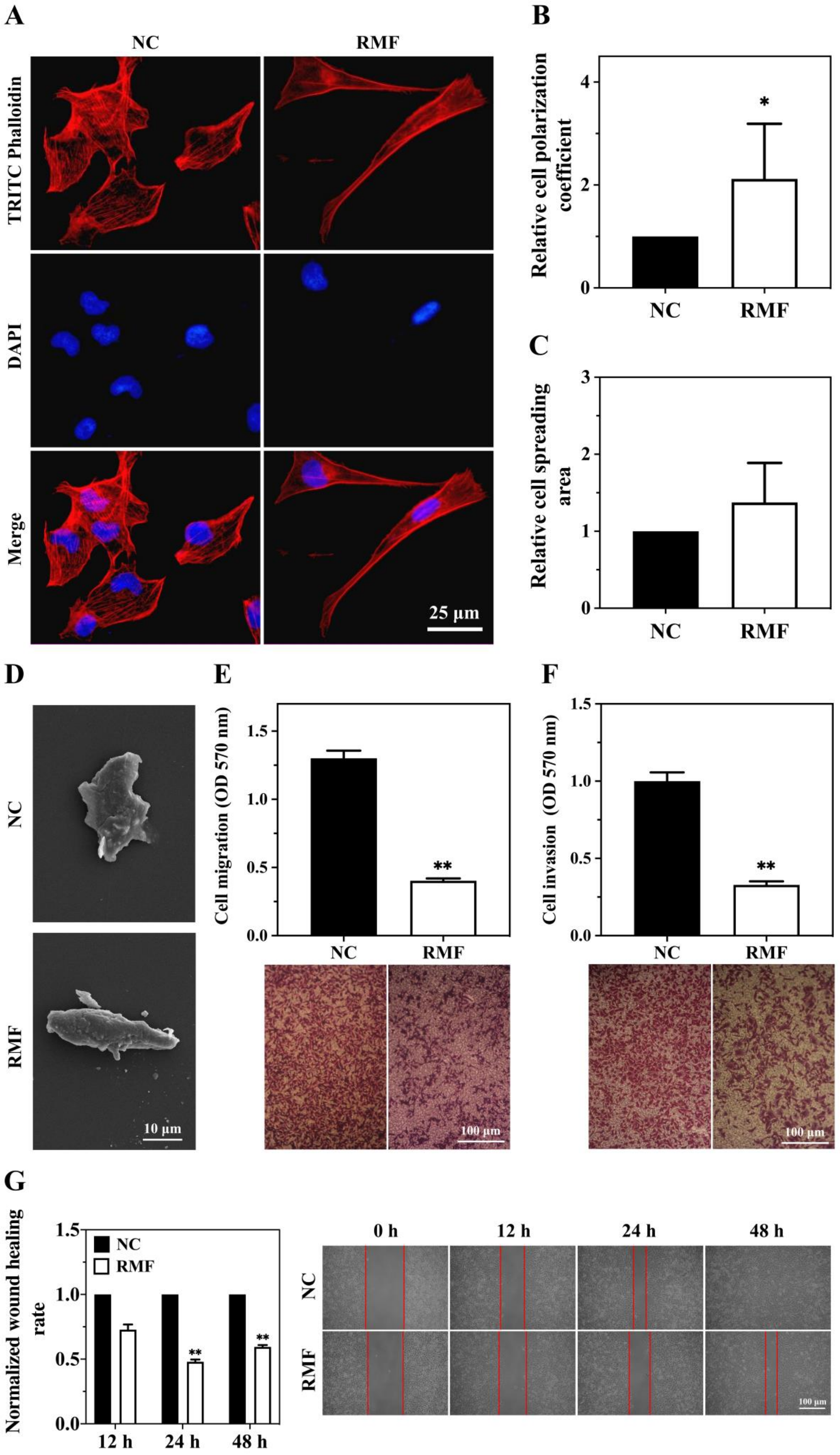
A
NC
RMF
TRITC Phalloidin
DAPI
Merge
25 µm
B
Relative cell polarization coefficient
*
NC
RMF
C
Relative cell spreading area
NC
RMF
D
NC
RMF
10 µm
E
Cell migration (OD 570 nm)
**
NC
RMF
100 µm
F
Cell invasion (OD 570 nm)
**
NC
RMF
100 µm
G
Normalized wound healing rate
NC
RMF
12 h
24 h
48 h
0 h
100 µm

Figure S3. Effect of gradient RMF on F-actin, migration, invasion, and wound healing capacity of MDA-MB-231 cells at 2.5 Hz. Effect of gradient RMF exposure on cytoskeleton (A), polarization coefficient (B), spreading area (C), morphology (D), migration (E), invasion (F) and wound healing capacity (G) of MDA-MB-231 cells at 2.5 Hz. n=3. Statistical analyses were conducted using *t*-test and one-way ANOVA. *$p < 0.05$, **$p < 0.01$, and ***$p < 0.001$ vs. NC group.

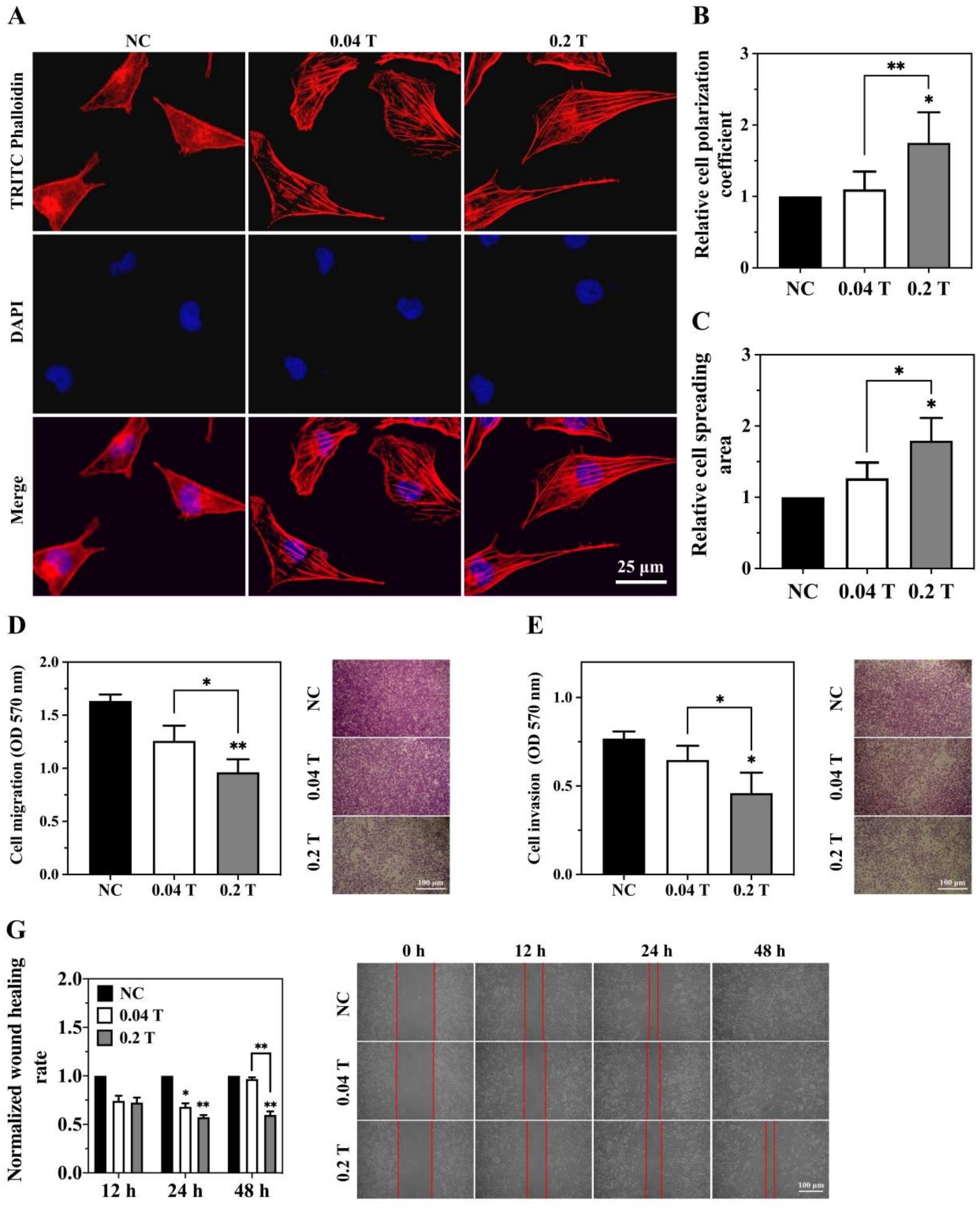

Figure S4. Effect of gradient RMF on F-actin, migration, invasion, and wound healing capacity of MDA-MB-231 cells at different inductions of magnetic field. Effect of gradient RMF exposure on cytoskeleton (A), polarization coefficient (B), spreading area (C), migration (D), invasion (E) and wound healing ability (F) of MDA-MB-231 cells at 0.04 T and 0.2 T, respectively. n=3. Statistical analyses were conducted using *t*-test, one-way or two-way ANOVA, and *post-hoc* tests were carried out. *$p < 0.05$, **$p < 0.01$, and ***$p < 0.001$ vs. NC group.

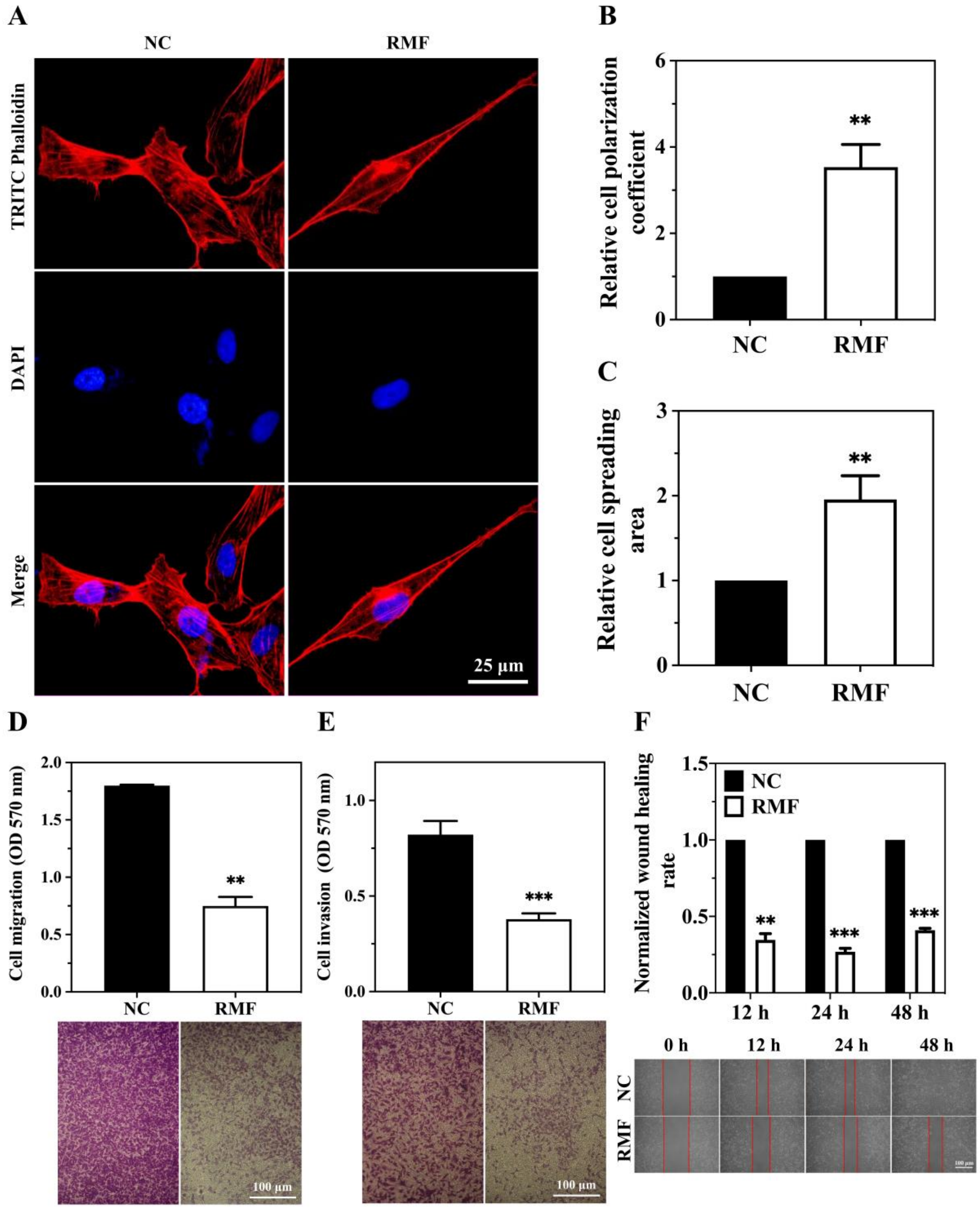

Figure S5. Effect of gradient RMF on F-actin, migration, invasion, and wound healing capacity of BT549 cells at optimal characteristics. Effect of gradient RMF exposure on cytoskeleton (A), polarization coefficient (B), spreading area (C), migration (D), invasion (E) and wound healing capacity of BT549 cells at 0.41 T, 5 Hz. n=3. Statistical analyses were conducted using *t*-test and one-way ANOVA. *$p < 0.05$, **$p < 0.01$, and ***$p < 0.001$ vs. NC group.

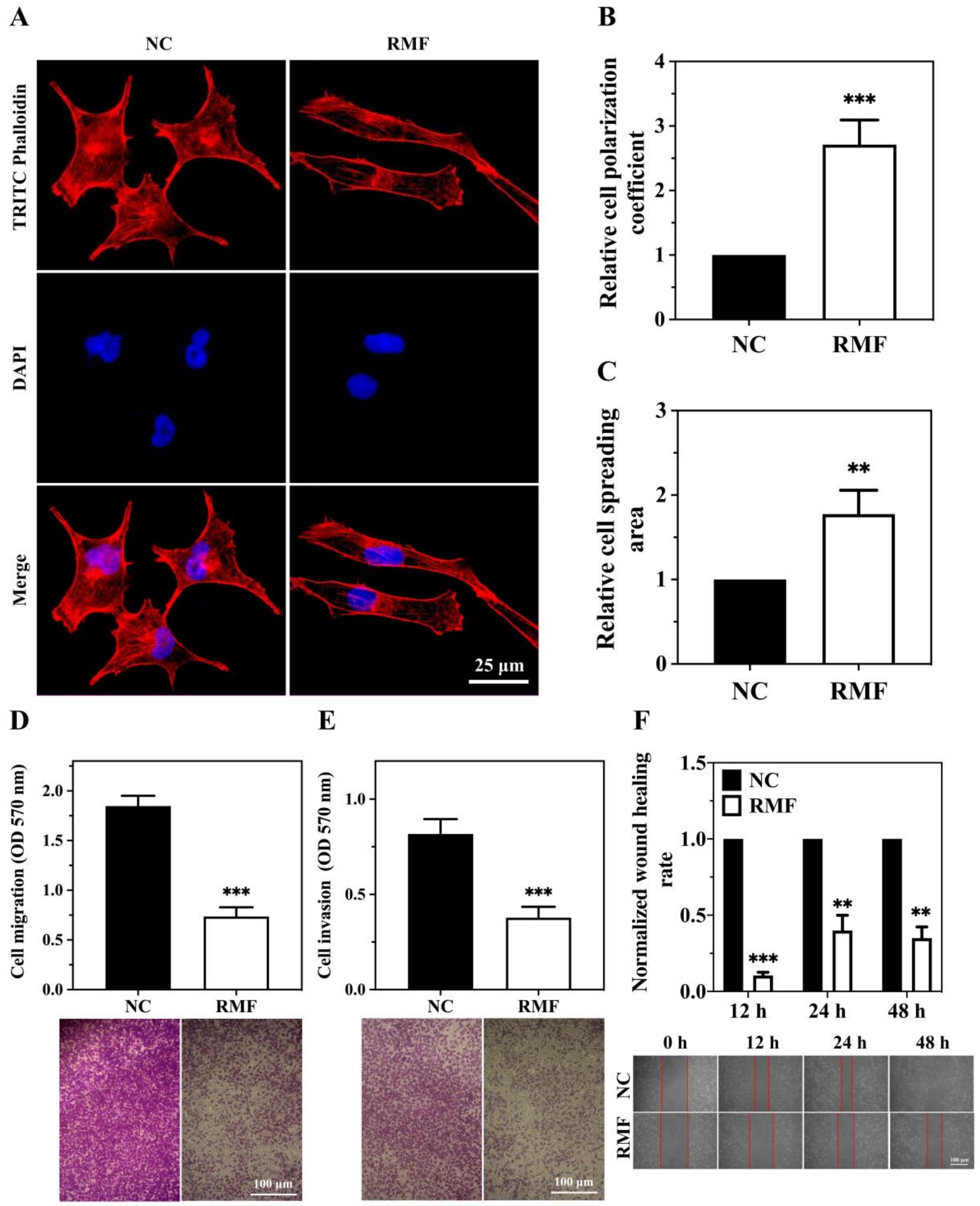

Figure S6. Effect of gradient RMF on the F-actin, migration, invasion, and wound healing capacity of MDA-MB-468 cells at optimal characteristics. Effect of gradient RMF exposure on cytoskeleton (A), polarization coefficient (B), and spreading area (C), migration (D), invasion (E) and wound healing capacity (F) of MDA-MB-468 cells at 0.41 T, 5 Hz. n=3. Statistical analyses were conducted using *t*-test and one-way ANOVA. *$p < 0.05$, **$p < 0.01$, and ***$p < 0.001$ vs. NC group.

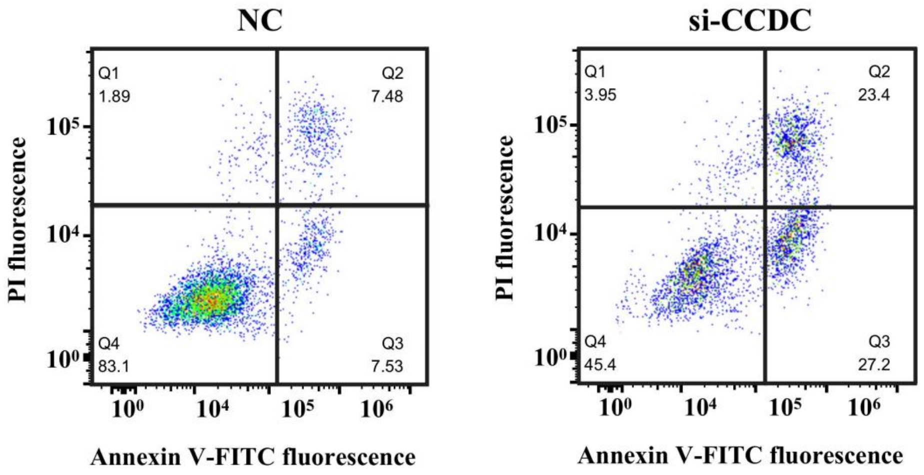

Figure S7. Effects of CCDC150 knocking-down on MDA-MB-231 cell apoptosis.

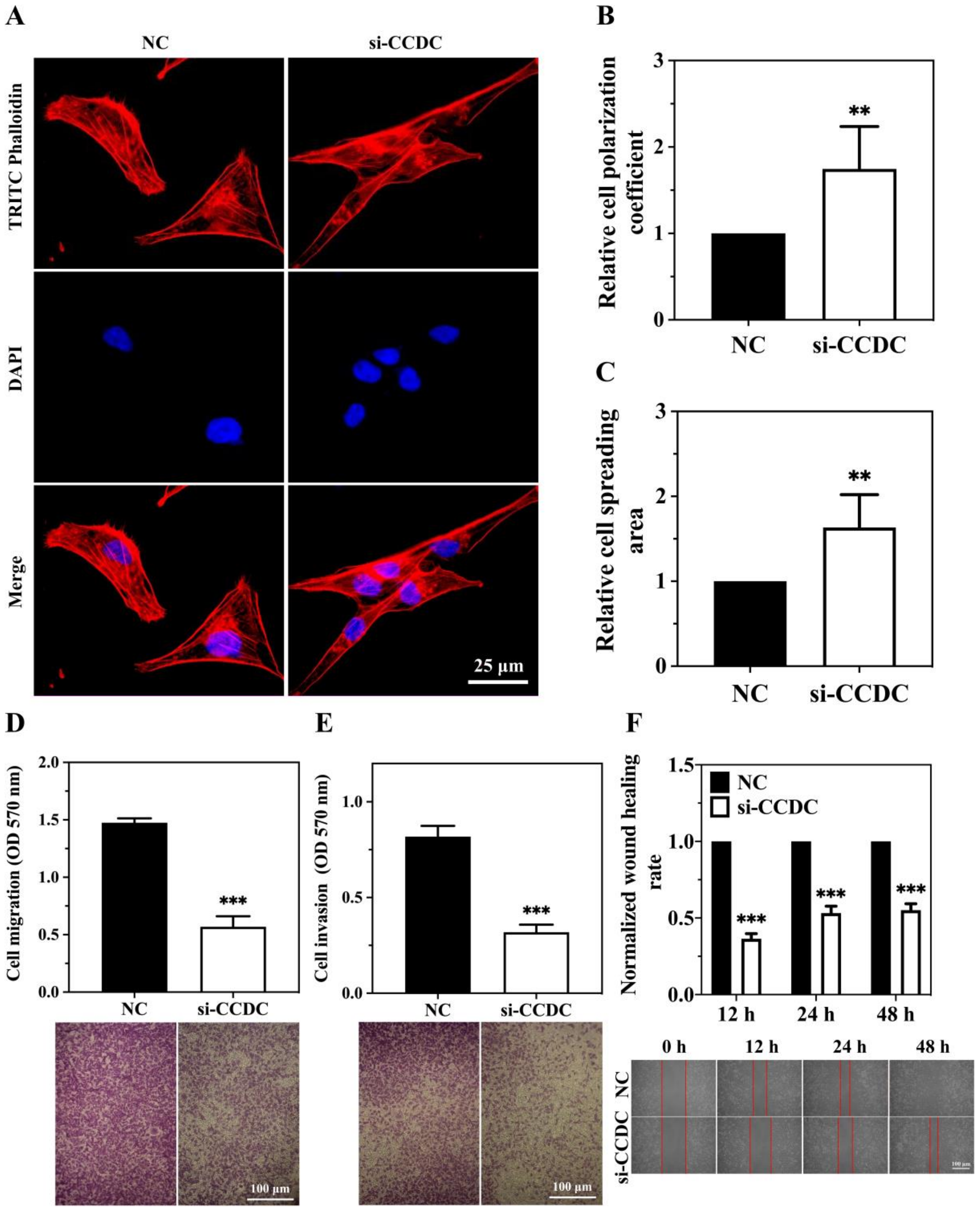

Figure S8. Effect of CCDC150 on F-actin, migration, invasion, and wound healing capacity of BT549 cells. Effect of CCDC150 knocking down on cytoskeleton (A), polarization coefficient (B), spreading area (C), migration (D), invasion (E) and wound healing capacity (F) of BT549 cells. n=3. Statistical analyses were conducted using *t*-test and one-way ANOVA. *$p < 0.05$, **$p < 0.01$, and ***$p < 0.001$ vs. NC group.

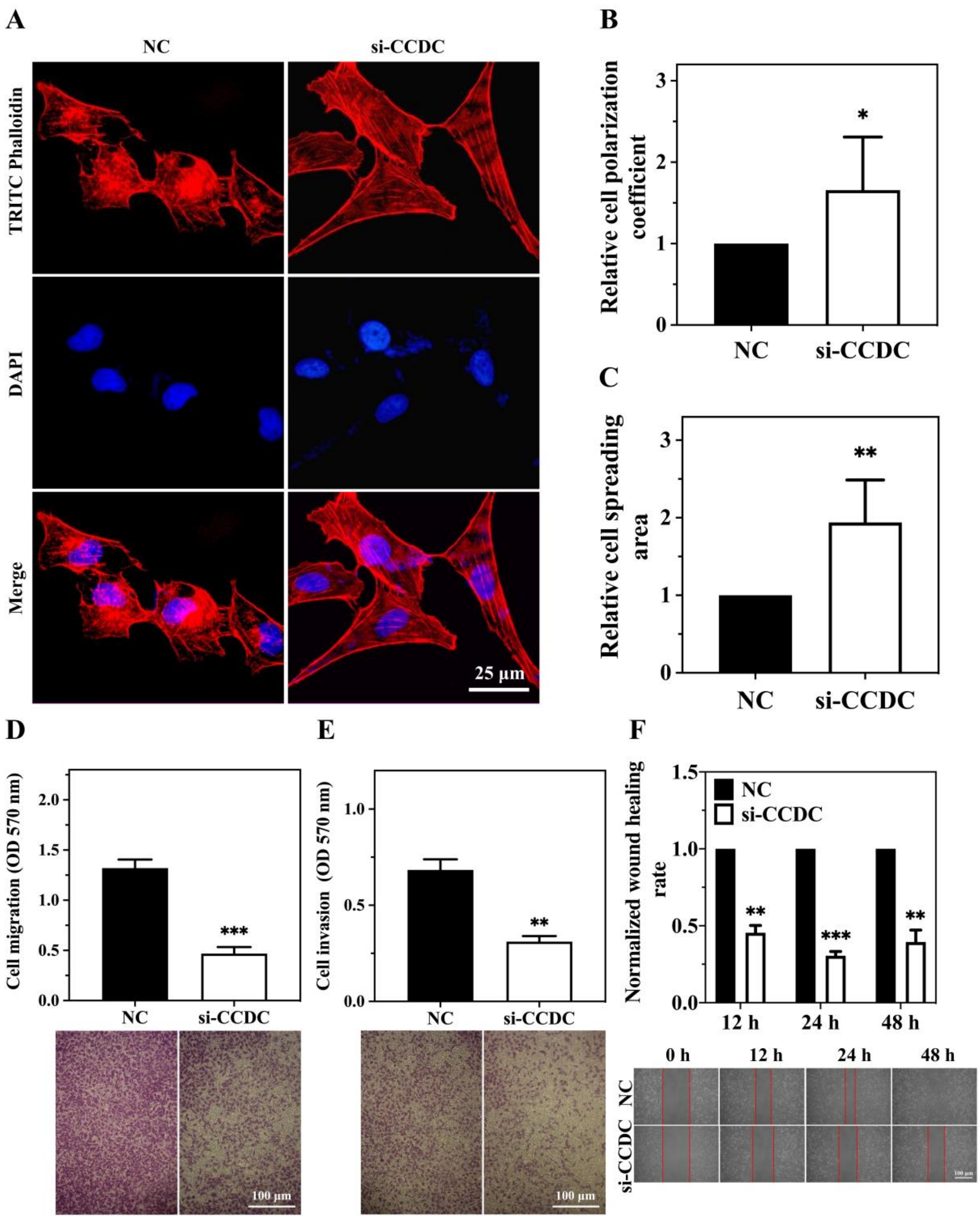

Figure S9. Effect of CCDC150 on F-actin, migration, invasion, and wound healing capacity of MDA-MB-468 cells. Effect of CCDC150 knocking down on cytoskeleton (A), polarization coefficient (B), spreading area (C), migration (D), invasion (E) and wound healing capacity (F) of MDA-MB-468 cells. n=3. Statistical analyses were conducted using *t*-test and one-way ANOVA. *p < 0.05, **p < 0.01, and ***p < 0.001 vs. NC group.

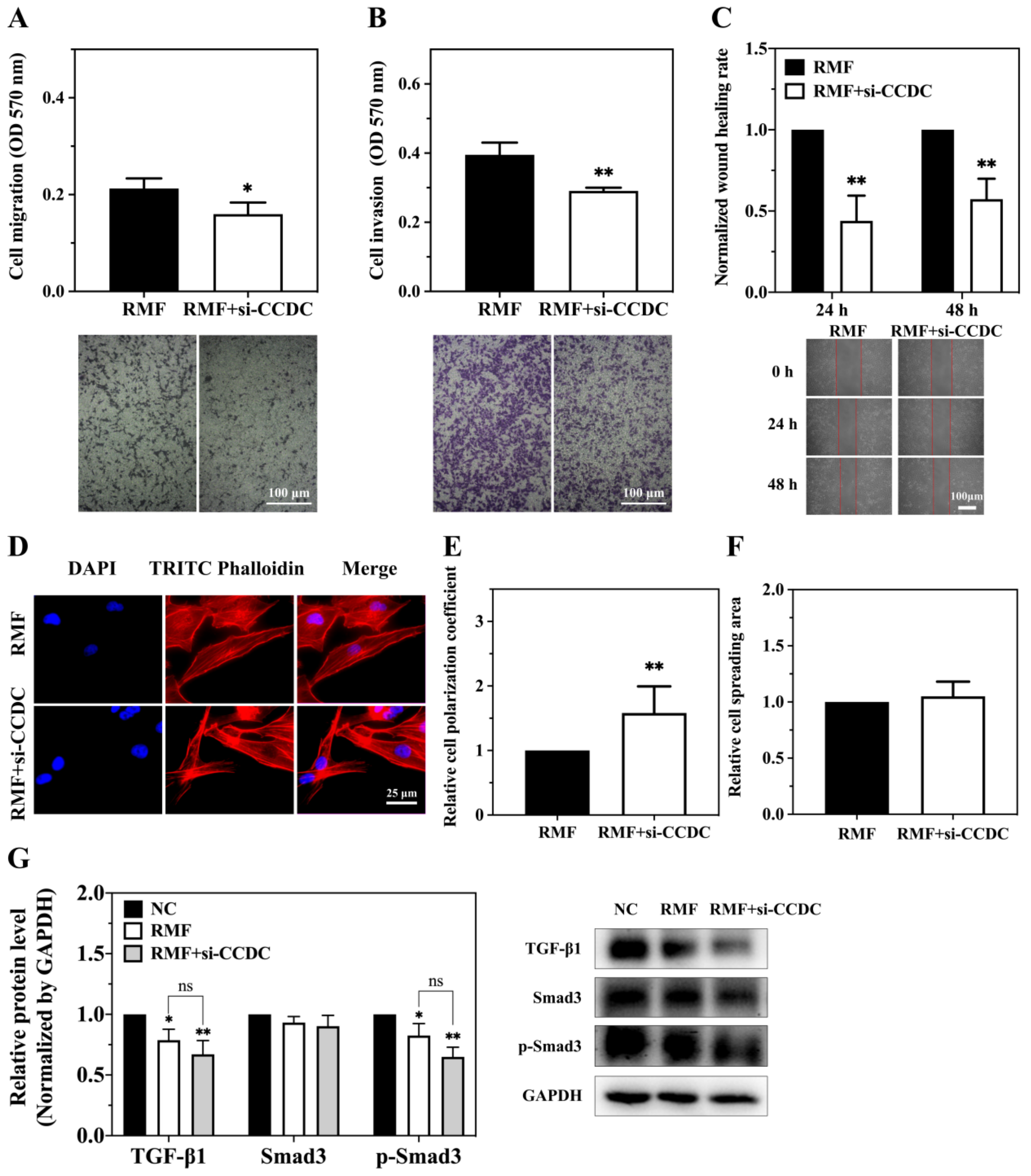

Figure S10. Effects of gradient RMF exposure or gradient RMF+si-CCDC combination treatment on MDA-MB-231 cells. Effect of gradient RMF or gradient RMF+si-CCDC on MDA-MB-231 cell migration (A), invasion (B) and wound healing capacity (C) at 0.41 T, 5 Hz. (D) Fluorescent staining of cytoskeleton F-actin, and its cell polarization coefficient (E) and spreading area (F) after treated with gradient RMF or gradient RMF+si-CCDC. (G) Expressions of TGF-β1/SMAD3 signaling pathway biomarkers. GAPDH was used as the reference gene. n=3. Statistical analyses were conducted using *t*-test, one-way or two-way ANOVA, and

post-hoc tests were carried out. *$p < 0.05$, **$p < 0.01$, and ***$p < 0.001$ vs. NC group.

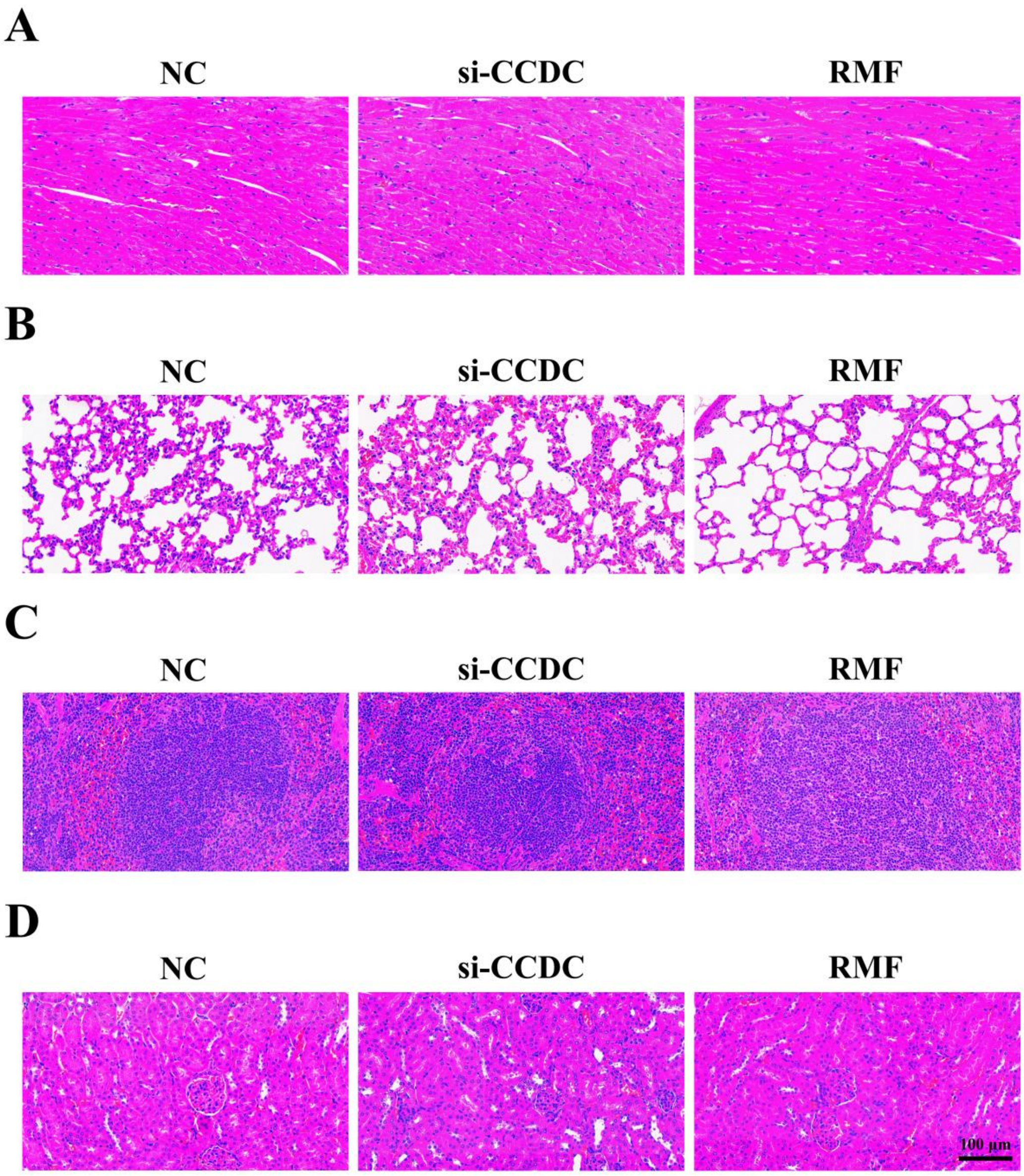

Figure S11. Effects of gradient RMF exposure or si-CCDC150 on the major organs of TNBC-bearing nude mice. H&E stained sections of heart (A), lung (B), spleen (C) kidney (D) of nude mice.

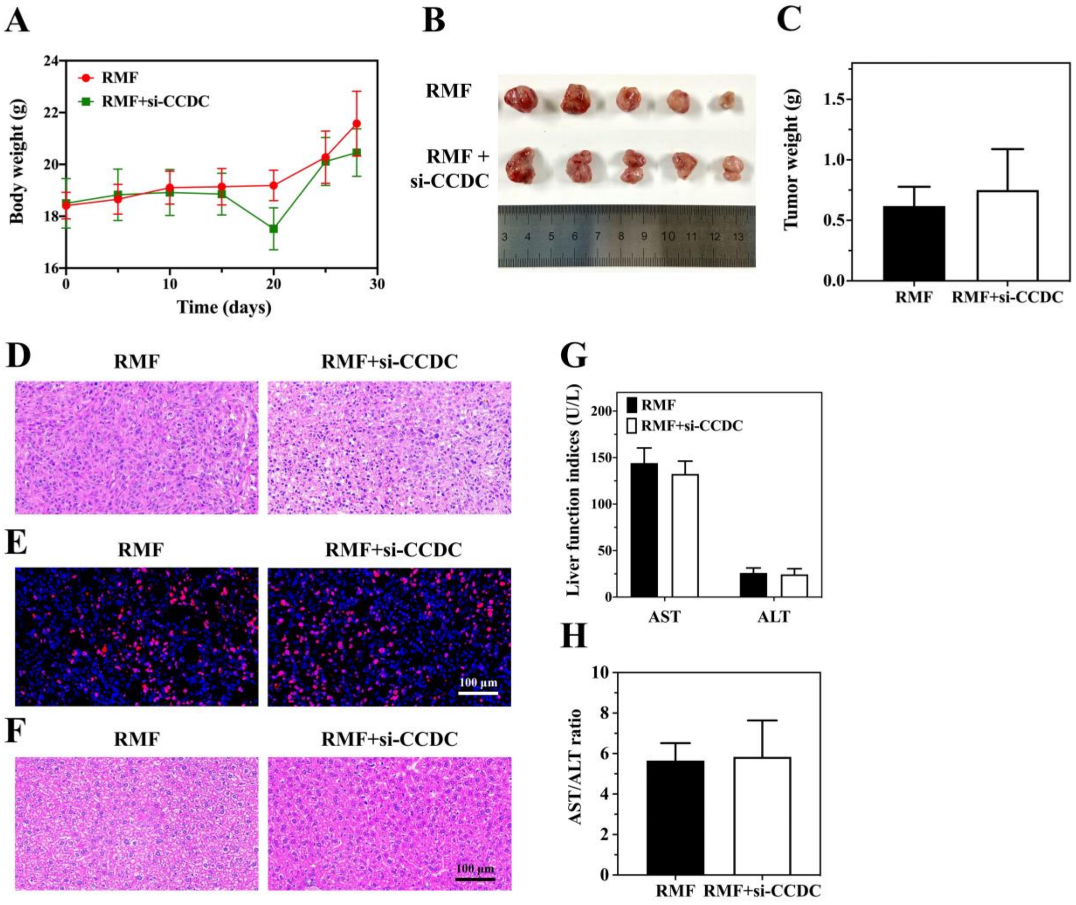

Figure S12. Effect of gradient RMF exposure or gradient RMF+siCCDC150 combination treatment on TNBC tumor growth *in situ*. Body weight (A), tumor image (B), and tumor weight (C) in gradient RMF exposure and gradient RMF+si-CCDC150 group. H&E (D) and Ki67 (E) staining of tumor tissue. (F) H&E staining section of liver. ALT and AST level (G) and ALT/AST ratio (H) in the serum of different treatment group. n=5. Statistical analyses were conducted using *t*-test and one-way ANOVA. *$p < 0.05$, **$p < 0.01$, and ***$p < 0.001$ vs. NC group.

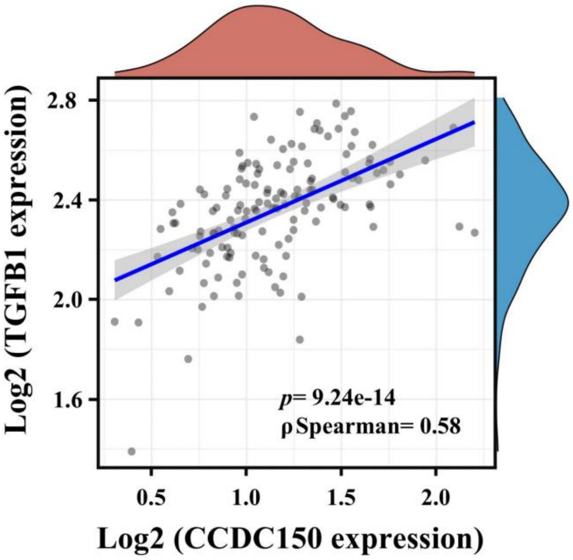

Figure S13. ρSpearman correlation analysis of CCDC150 and TGF-β1.

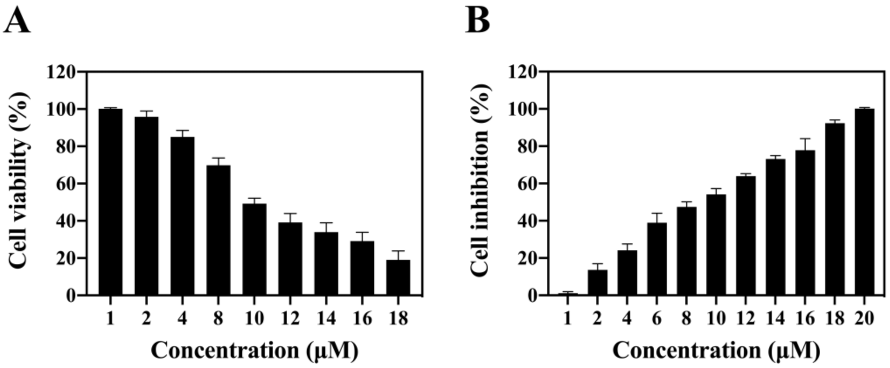

Figure S14. SD208 and SRI-011381 treatment concentration screening. IC50 of SD208 (A) and EC50 of SRI-011381 (B).

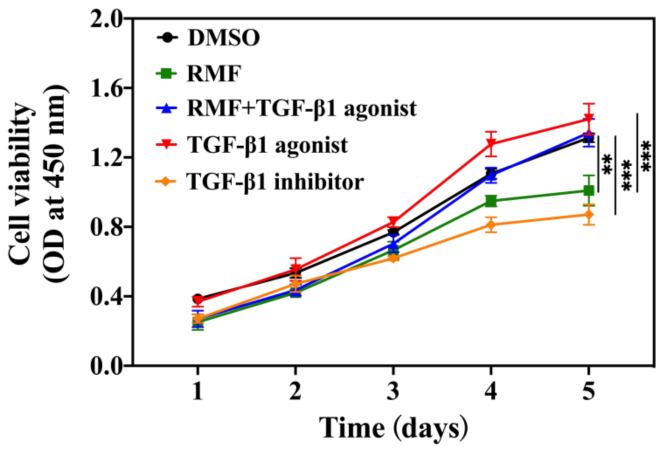

Figure S15. Effect of gradient RMF and TGF-β1/SMAD3 signaling pathway on the MDA-MB-231 cell viability. Statistical analysis was performed using one-way or two-way ANOVA, followed by *post-hoc* tests. n = 3. *$p < 0.05$ and **$p < 0.01$ vs. DMSO group.
