# Supplementary figures and images for: Gradient Rotating Magnetic Fields Impairing F-Actin-Related Gene CCDC150 to Inhibit Triple-Negative Breast Cancer Metastasis by Inactivating TGF-β1/SMAD3 Signaling Pathway
Source: Research (Wash D C). 2024 Feb 28;7:0320. doi: 10.34133/research.0320 (PMC10900498; doi:10.34133/research.0320)

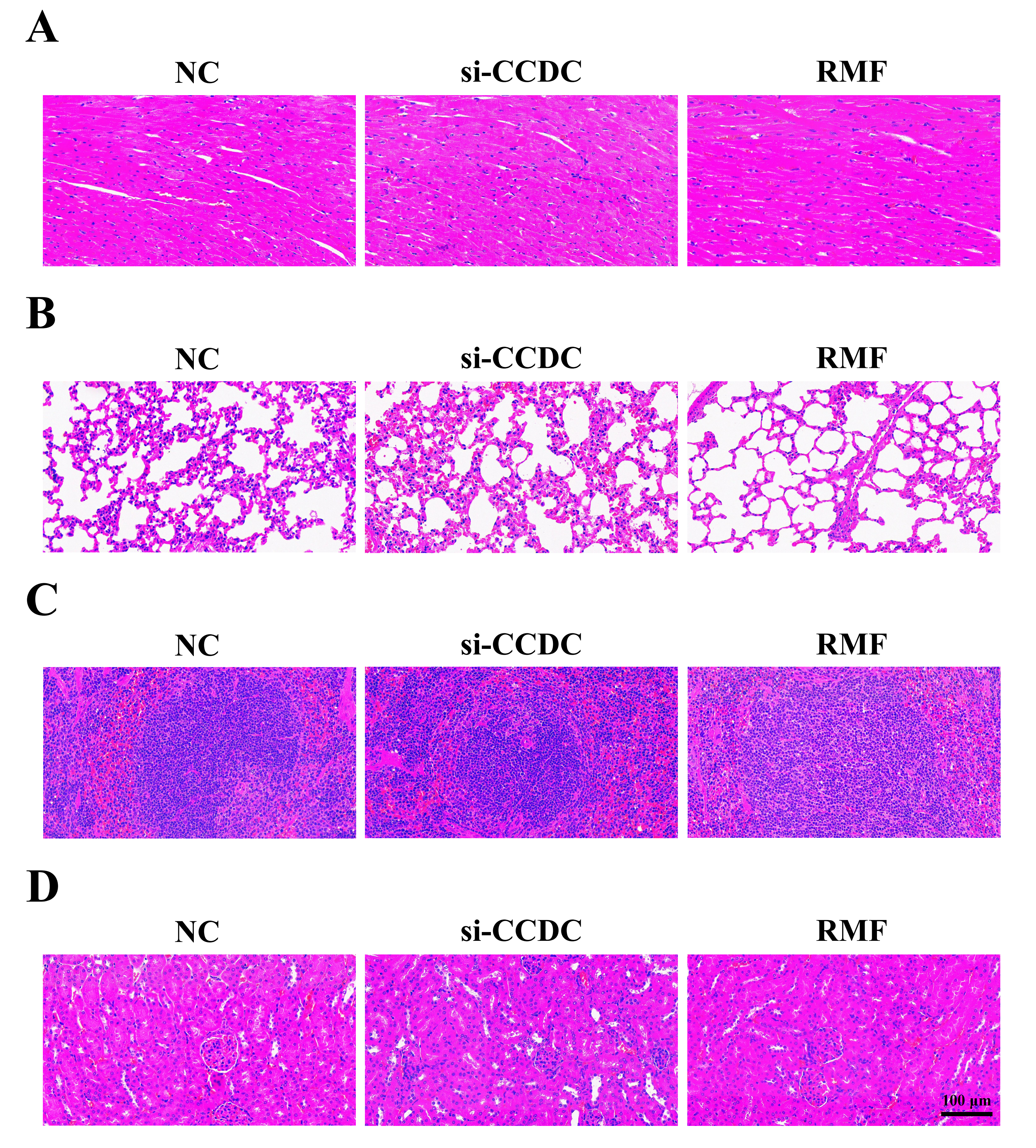

Supplement: Supplementary 1 — Figs. S1 to S15 Tables S1 and S2 [file research.0320.f1.zip › Figure S11.png]

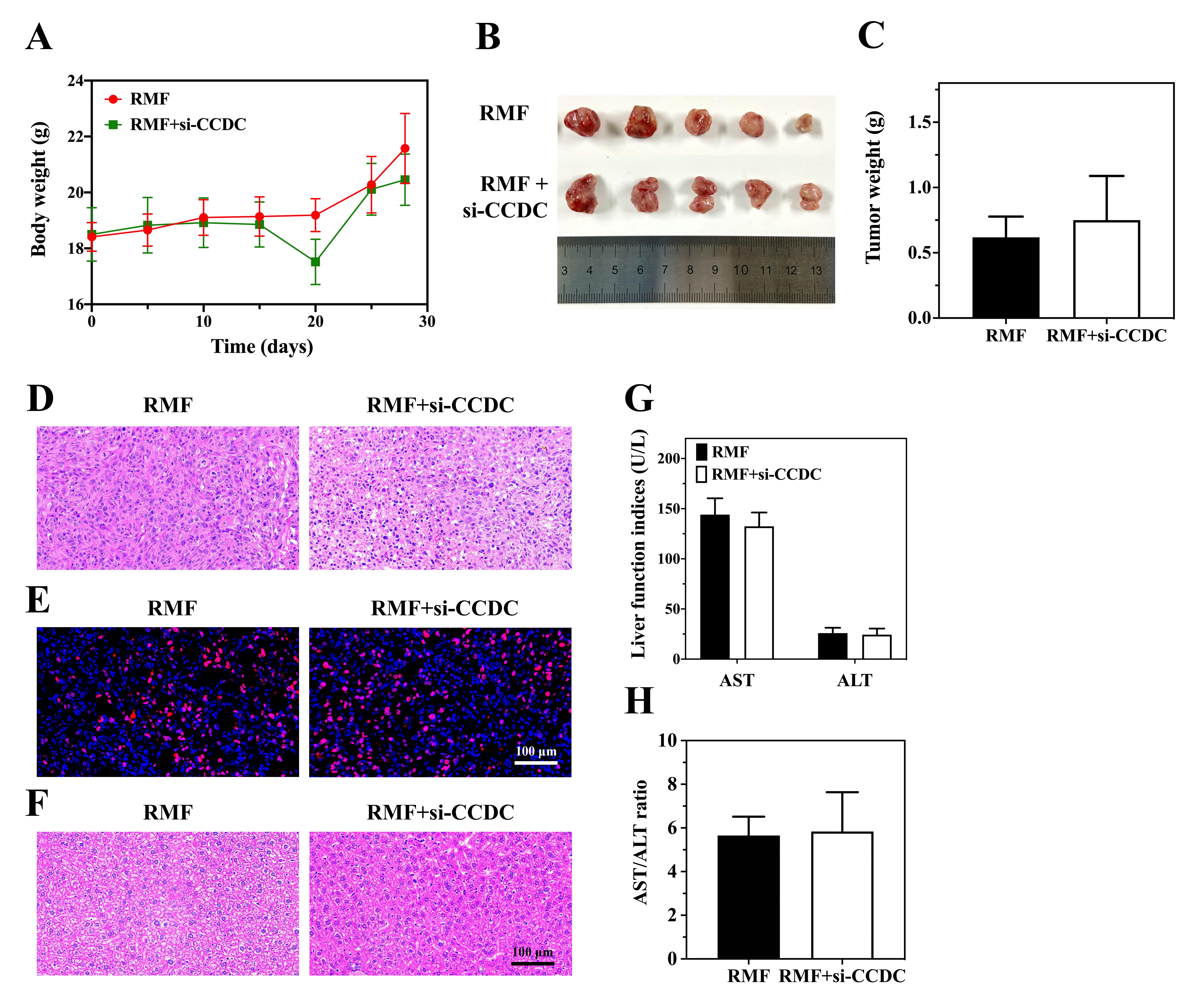

Supplement: Supplementary 1 — Figs. S1 to S15 Tables S1 and S2 [file research.0320.f1.zip › Figure S12.png]

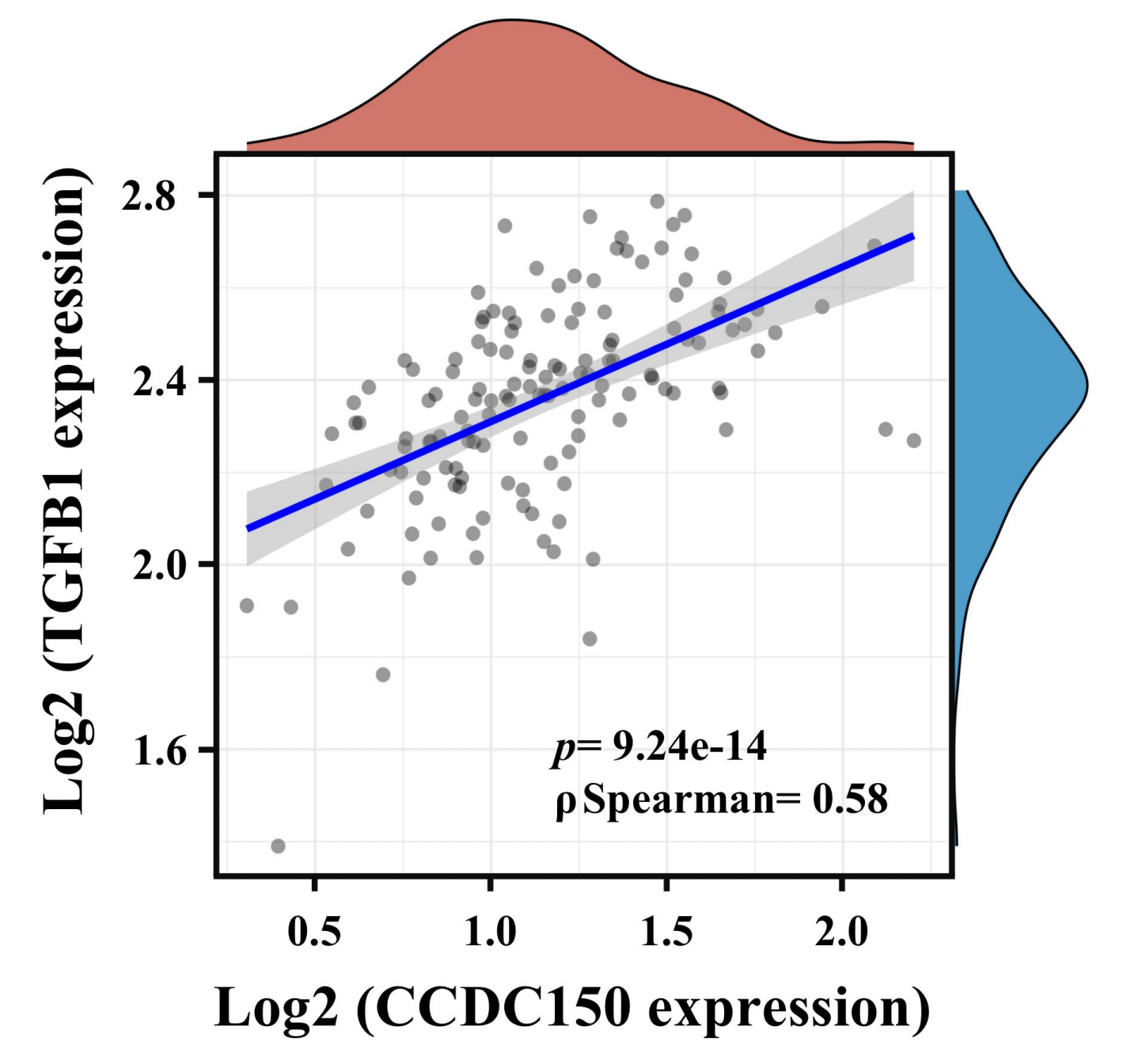

Supplement: Supplementary 1 — Figs. S1 to S15 Tables S1 and S2 [file research.0320.f1.zip › Figure S13.png]

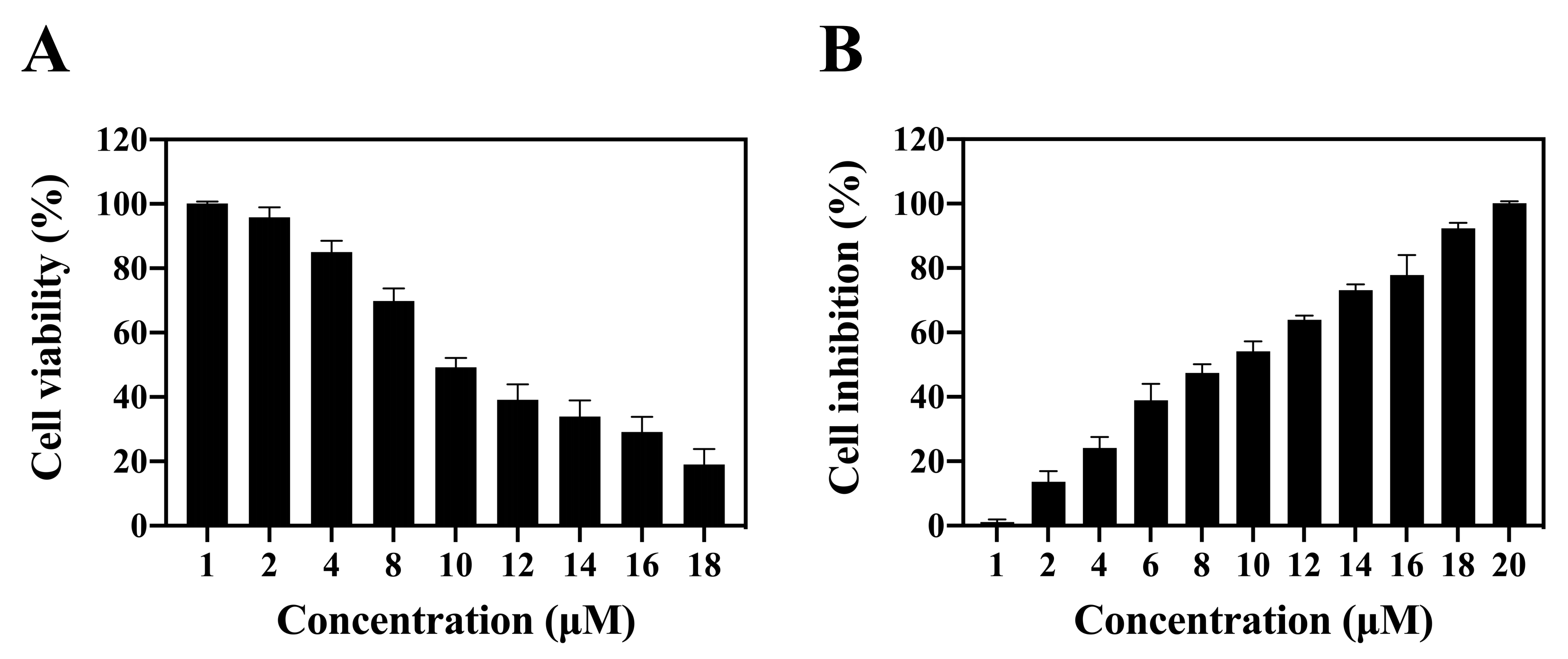

Supplement: Supplementary 1 — Figs. S1 to S15 Tables S1 and S2 [file research.0320.f1.zip › Figure S14.png]

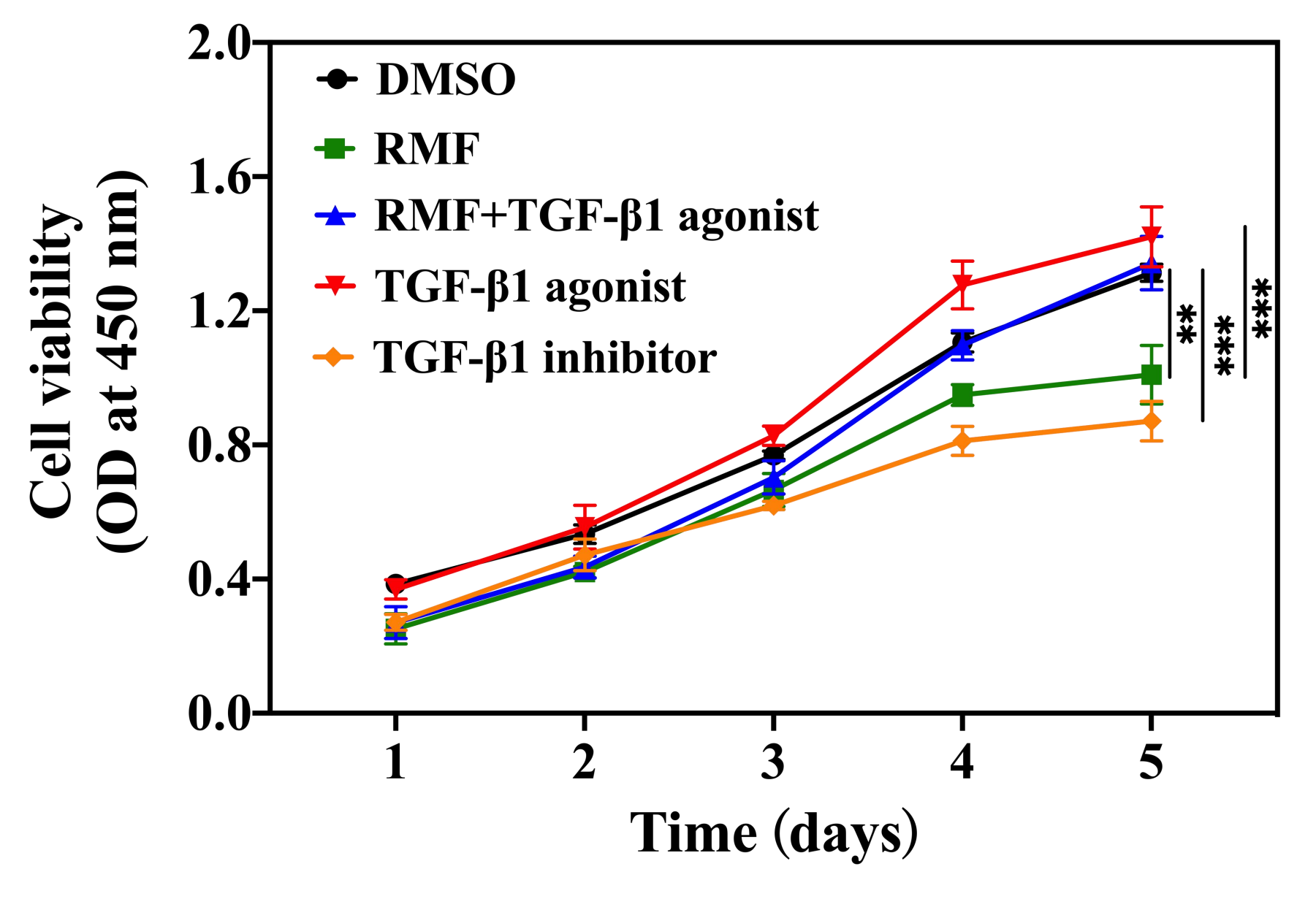

Supplement: Supplementary 1 — Figs. S1 to S15 Tables S1 and S2 [file research.0320.f1.zip › Figure S15.png]

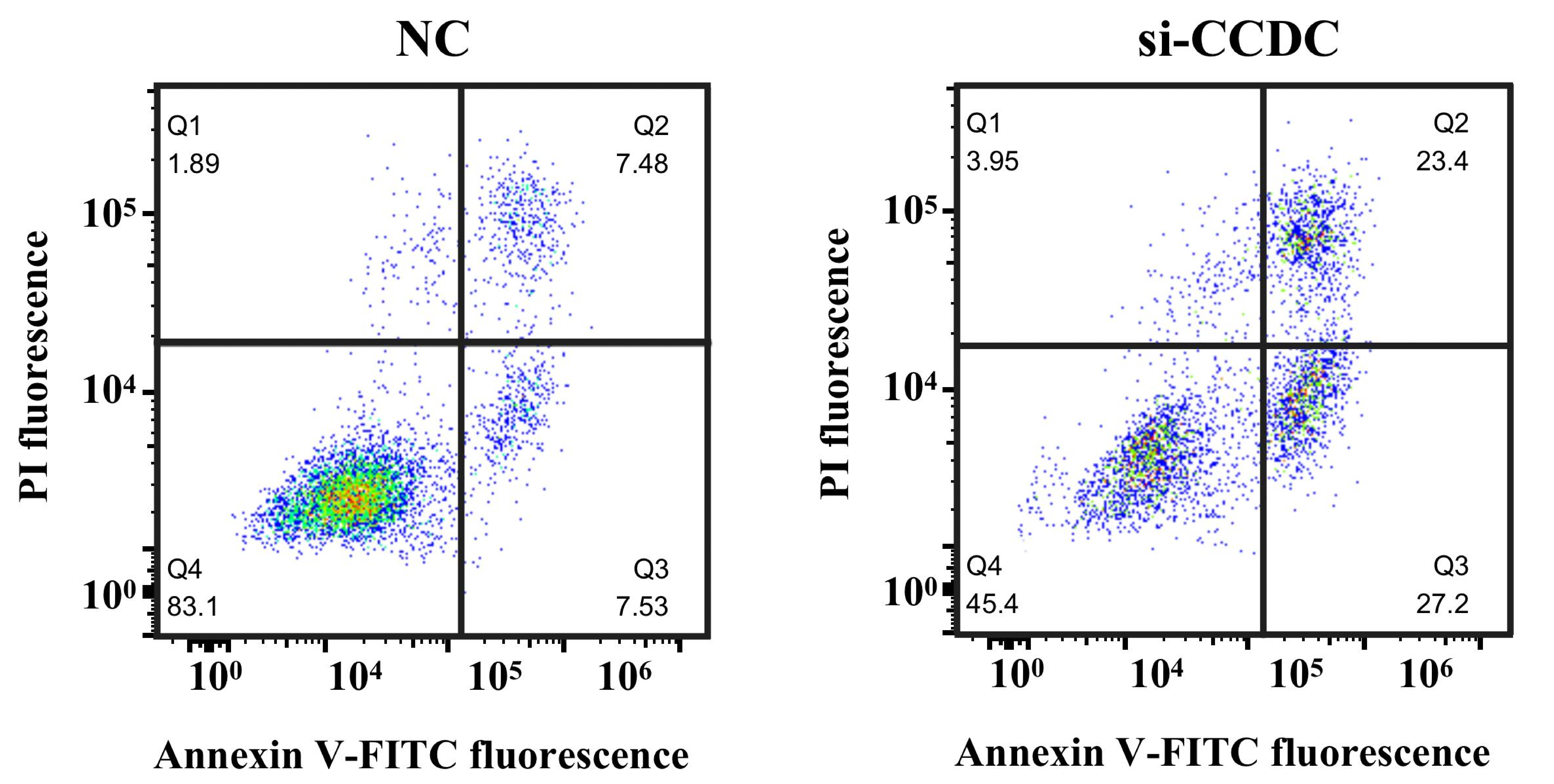

Supplement: Supplementary 1 — Figs. S1 to S15 Tables S1 and S2 [file research.0320.f1.zip › Figure S7.png]
